# Whole-Genome and Transcriptome Sequencing of *Phlebopus portentosus* Reveals Its Associated Ectomycorrhizal Niche and Conserved Pathways Involved in Fruiting Body Development

**DOI:** 10.3389/fmicb.2021.732458

**Published:** 2021-09-29

**Authors:** Jia-Ning Wan, Yan Li, Ting Guo, Guang-Yan Ji, Shun-Zhen Luo, Kai-Ping Ji, Yang Cao, Qi Tan, Da-Peng Bao, Rui-Heng Yang

**Affiliations:** ^1^Key Laboratory of Agricultural Genetics and Breeding of Shanghai, Key Laboratory of Edible Fungal Resources and Utilization (South), National Engineering Research Center of Edible Fungi, Institute of Edible Fungi, Shanghai Academy of Agricultural Sciences, Shanghai, China; ^2^Hongzhen Agricultural Science and Technology Co. Ltd., Jinghong, China

**Keywords:** ectomycorrhizal fungi, genome, transcriptome, CAZymes, plant cell wall degradation

## Abstract

Phlebopus *portentosus* (Berk. and Broome) Boedijin, a widely consumed mushroom in China and Thailand, is the first species in the order Boletaceae to have been industrially cultivated on a large scale. However, to date, the lignocellulose degradation system and molecular basis of fruiting body development in *P. portentosus* have remained cryptic. In the present study, genome and transcriptome sequencing of *P. portentosus* was performed during the mycelium (S), primordium (P), and fruiting body (F) stages. A genome of 32.74 Mb with a 48.92% GC content across 62 scaffolds was obtained. A total of 9,464 putative genes were predicted from the genome, of which the number of genes related to plant cell wall-degrading enzymes was much lower than that of some saprophytic mushrooms with specific ectomycorrhizal niches. Principal component analysis of RNA-Seq data revealed that the gene expression profiles at all three stages were different. The low expression of plant cell wall-degrading genes also confirmed the limited ability to degrade lignocellulose. The expression profiles also revealed that some conserved and specific pathways were enriched in the different developmental stages of *P. portentosus*. Starch and sucrose metabolic pathways were enriched in the mycelium stage, while DNA replication, the proteasome and MAPK signaling pathways may be associated with maturation. These results provide a new perspective for understanding the key pathways and hub genes involved in *P. portentosus* development.

## Introduction

*Phlebopus portentosus* (Berk. and Broome) Boedijin is an ectomycorrhizal edible mushroom with a widespread distribution in tropical parts of China, especially Yunnan, Guangxi, and Hainan provinces (Zhang et al., 2017). Because it is rich in nutrients, e.g., polysaccharides, amino acids, mineral elements, and pyrrole alkaloids, this fungus is popular and widely used in these regions (Sanmee et al., 2010; Sun et al., 2018; Kumla et al., 2021). *P. portentosus* has been successfully artificially cultivated and produces sporocarps in artificial substances *in vitro* in greenhouses or factories in China and Thailand, making it the first species in the order Boletaceae to have been industrially cultivated on a large scale (Ji et al., 2011; Kumla et al., 2015; Zhang et al., 2017). Especially in China, the industrialized cultivation of *P. portentosus* is highly similar to the *Agaricus* mushroom industry, with a daily production of 6 tons (Zhang et al., 2017). Nutrition and management techniques play important roles in maintaining the yields and quality of mushrooms (Tseng and Luong, 1984). Furthermore, understanding the lignocellulose degradation system and fruiting body development of this fungus is crucial to enhance yields and improving quality (Isikhuemhen and Mikiashvilli, 2009). However, the molecular basis of these biological processes is not fully understood.

Although *P. portentosus* can be cultivated by providing carbon or nitrogen compounds in artificial media, it is still characterized as an ectomycorrhizal (ECM) fungus that requires the use of coculture and isotopic methods (Pham et al., 2012; Kumla et al., 2016). Because most ectomycorrhizal fungi have difficulty producing macroscopic sporocarps in axenic culture on a large scale (Hall et al., 2003), some studies have revealed that two ecological niches may be required for *P. portentosus* growth, both independently as a saprophyte and in association with plants as an ectomycorrhizal symbiont, offering an excellent model to study the mechanisms by which fungi obtain nutrition from *in vitro* environments (Cao et al., 2015; Zhang et al., 2017). Plant cell wall-degrading enzymes (PCWDEs) secreted by fungi, including cellulases, hemicellulases, pectinases, and ligninases, provide fungi with the means to acquire energy and nutrients from plant cell walls (Hammel, 1997). However, genomic and transcriptomic comparisons between ectomycorrhizal and saprophytic fungi have revealed a reduced repertoire of genes encoding PCWDEs in ectomycorrhizal fungi, resulting in a limited capacity to decompose lignocellulose (Kohler et al., 2015; Miyauchi et al., 2020). A genome analysis of *P. portentosus* also revealed the presence of few or no copies of carbohydrate metabolism enzymes (CAZymes) that act on cellulose, xylan, pectin, and lignin (Cao et al., 2015). It is surprising that so few genes for lignocellulose degradation support *P. portentosus* growth on sawdust substances. However, our understanding of these enzymatic repertoires of *P. portentosus* is far from complete.

The hyphal differentiation, fruiting body formation and development of edible mushrooms are complex processes. The expression of genes at different stages of fungal development (hyphae, primordia, and fruiting body) is associated with yield and quality. Thus, an understanding of the molecular mechanisms regulating fruiting body development is necessary to generate improved strains and varieties of mushrooms by genetic modification or breeding. With the advent of next-generation sequencing techniques, genome and transcriptome sequencing of saprophytic and ectomycorrhizal mushrooms has been widely used to assess gene expression in different growth stages, including for *Agrocybe aegerita* (Wang et al., 2013), *Lentinula edodes* (Song et al., 2018; Wang et al., 2018), *Flammulina velutipes* (Liu et al., 2020), *Pleurotus eryngii* (Xie et al., 2018), *Morchella* (Hao et al., 2019; Wei et al., 2019), and *Tricholoma matsutake* (Tang et al., 2020). Stage-specific pathways enriched and correlated with the growth and development of fruiting bodies have been identified, with functions related to fungal cell wall remodeling, targeted protein degradation, signal transduction, adhesion, and small secreted proteins. Although *P. portentosus* has been successfully artificially cultivated, gene expression in this fungus grown *in vitro* during different developmental stages is poorly understood.

To better understand the molecular mechanism of lignocellulose degradation and fruiting body development, whole-genome and transcriptome sequencing of *P. portentosus* was performed. Three developmental stages were analyzed, including hyphal, primordia, and fruit body stages. The results of genomic and transcriptomic analyses will improve our understanding of the stage-specific expression of genes associated with the functional properties of fruiting body development in *P. portentosus*. In addition, the transcriptomic profiles of ectomycorrhizal (ECM) fungi cultivated *in vitro* provide a foundation for further research on the cultivation of other ectomycorrhizal (ECM) fungi.

## Materials and Methods

### Strain Collection, Artificial Cultivation and Monokaryotic Strain Preparation

The strain PP17026, which was collected from Yunnan Province, China, was provided by Hongzhen Agricultural Science and Technology Co. Ltd. and cultured on M1 medium in the dark at 30°C. Spawn preparation, artificial cultivation and management of *P. portentosus* was performed according to previously published methods (Yang et al., 2019). Samples were collected in the hyphal (S, stage III), primordia (P, stage VI), and fruiting body (F, stage VII) stages (Yang et al., 2019). The hyphae fully covered on the surface of substrates, the small primordia with about 0.2 cm height and the stipes and pileus (cut into 0.2 cm × 0.2 cm pieces) used as representative samples of S, P, and F stages respectively were collected and flash-frozen in liquid nitrogen and stored at –80°C before RNA extraction.

The monokaryotic protoplasts were isolated from PP17026 using a previously published method (Chang et al., 1985). After 10 days of incubation, mycelia were collected and stored at –80°C until DNA and RNA extraction for genome and transcriptome sequencing. Transcriptome sequencing of monokaryotic strains was performed to correct the gene prediction results from the genome annotation.

### DNA Extraction, Library Construction and Genome Sequencing

DNA was extracted from the monokaryotic strain using the CTAB method. A total of 100–200 mg wet weight mycelium was ground in liquid nitrogen using a pestle in a centrifugal tube. Then, 1.5 mL of extraction buffer was added containing 20 mM EDTA, 100 mM Tris-HCl, 1.5 M NaCl, 2% CTAB, and 1% β-mercaptoethanol, and the sample was incubated at 65°C for 30 min. Protein and polysaccharide removal and DNA precipitation were conducted using chloroform-isoamyl alcohol (24:1, v/v) and isopropanol, respectively. The concentration, purity and integrity of DNA were assessed by NanoDrop (Thermo Scientific), Qubit and pulsed field electrophoresis analysis, respectively. A 20-kb sequencing library was built using large DNA segments with an ONT Template prep kit (SQK-LSK109) and an NEB Next FFPE DNA Repair Mix kit. The high-quality library was sequenced on an ONT PromethION platform with a corresponding R9 cell and an ONT sequencing reagent kit (EXP-FLP001.PRO.6). In addition, a small 300-bp sequencing library was built for the Illumina platform to improve the accuracy of the long-fragment library sequencing results.

### RNA Extraction, Library Construction and RNA Sequencing

Total RNA were extracted from all samples as previously described using TRIzol reagent (Invitrogen, Burlington, ON, Canada). Three biological replicates were processed. The purity and concentration of RNA was assessed using a NanoPhotometer^®^ spectrophotometer (IMPLEN, CA, United States) and a Qubit^®^ RNA Assay Kit with a Qubit^®^2.0 Flurometer (Life Technologies, CA, United States). RNA integrity was assessed using an RNA Nano 6000 Assay Kit with the Agilent Bioanalyzer 2100 system (Agilent Technologies, CA, United States). Subsequently, RNA was treated with DNase I (Thermo Fisher, Waltham, MA, United States), and the mRNA was purified based on PolyA selection and fragmentation. First strand cDNA synthesis was performed using SuperScript II (Thermo Fisher) followed by second strand cDNA synthesis, end repair, 30-end adenylation, adapter ligation and PCR amplification. Purification was performed using AmPureXP Beads (Beckman Coulter, Brea, CA, United States). The 300 bp libraries were sequenced for paired-end 150-bp-reads on Illumina HiSeq Novaseq 6000 platform (Illumina Inc., United States).

### Genome Assembly and Annotations

To retrieve the nucleotide sequences from raw signal data generated from the ONT platform, base calling was performed using Albacore implemented in MinKNOW (Sahoo, 2017). Subsequently, by filtering the low-quality reads and demultiplexing the ONT barcodes and short reads, the filtered subreads were obtained using Canu v1.5 (Sergey et al., 2017). Raw Illumina data trimming was performed according to previously published methods (Yang et al., 2016). The corrected subreads were assembled into contigs using wtdbg v1.2.8 (Ruan and Li, 2020). To improve assembly, contig correction with Illumina read data was performed using Pilon (Walker et al., 2014). The completeness of the genome assembly was evaluated using BUSCO 4.0 with fungi_odb9 (Simão et al., 2015). MicroRNAs, rRNAs, and tRNAs were identified using tRNAscan-SE (Lowe and Eddy, 1997) and Infernal 1.1 (Nawrocki, 2014).

Three methods were used for gene prediction: (i) using a combination of the results from Genscan (Burge and Karlin, 1998), Augustus v2.4 (Mario and Burkhard, 2005), GlimmerHMM v3.0.4 (Majoros et al., 2004), GeneID v1.4 (Blanco et al., 2002), and SNAP (version 2006-07-28) (Ian, 2004); (ii) homologous protein prediction conducted using GeMoMa v1.3.1 (Jens et al., 2019); and (iii) unigenes were assembled and predicted using Hisat2 v2.0.4 (Kim et al., 2019), StringTie v1.2.3 (Pertea et al., 2015) and TransDecoder v2.0 based on the transcriptome sequencing of monokaryotic strains. All three results were integrated using EVM v1.1.1 to obtain an accurate prediction (Haas et al., 2008). Predicted genes were annotated using BLAST (Basic Local Alignment Search Tool) searches against Swiss-Prot (Boeckmann et al., 2005), TrEMBL (Rédei, 2008) and Nr databases. Gene ontology and KEGG metabolic pathway matches were identified using local Blast2GO tools (Conesa et al., 2005) and KAAS (Yuki et al., 2007), respectively. All predicted protein families were analyzed with InterProScan (Quevillon et al., 2005) and Pfam analysis (Finn et al., 2010). Carbohydrate-active enzymes were annotated using dbCAN (Huang et al., 2018). Oxidoreductases were extracted from the proteins predicted from the genome using a combination of IPR domain searches and the JGI cluster pipeline^1^ according to a previously published method (Floudas et al., 2012). The A- and B-mating-type genes were identified using previously published methods (Chen et al., 2016). The genome has been submitted to the NCBI under the accession numbers of JAHRGP000000000.

### Data Processing, Alignment With the Reference Genome, Differentially Expressed Gene Analysis and Annotations for RNA Sequencing

The 150 bp paired-end reads obtained from the RNA-Seq analysis were trimmed using SeqPrep and Sickle to remove adaptor sequences, low-quality reads (those with ambiguous nucleotides and quality scores < 20). The filtered reads were mapped to the genome using HISAT2 v2.0.4. Transcripts were assembled and reconstructed using StringTie based on the HISAT2 mapping files. Several databases were used to annotate gene functions, including the Nr (NCBI non-redundant protein sequences), Nt (NCBI non-redundant nucleotide sequences), KOG/COG (Eukaryotic Ortholog Groups/Clusters of Orthologous Groups of proteins), Swiss-Prot (a manually annotated and reviewed protein sequence database), KO (KEGG Ortholog) and GO (Gene Ontology) databases. Gene expression was normalized using the FPKM (fragments per kilobase of transcript million mapped reads) method. The differentially expressed genes (DEGs) across all the samples were identified using DESeq2 (Love et al., 2014). Genes showing at least a twofold gene expression change with an FDR value < 0.05 were considered significantly differentially expressed. GO (Fisher’s exact test with corrected *P*-value < 0.05) and KEGG (Fisher’s exact test with corrected *P*-value < 0.05) enrichment analyses of DEGs associated with the significantly up- and downregulated genes between different stages were performed using Goatools (Klopfenstein et al., 2018) and R packages (Oksanen et al., 2011), respectively. Venn, PCA and hierarchical clustering for all correlation analyses were conducted using R packages. Weighted gene coexpression network analysis (WGCNA) was performed according to the method published previously (Li et al., 2019).

### Quantitative Reverse Transcription (RT)-Polymerase Chain Reaction (PCR) and Semiquantitative RT-PCR

For each sample, 1 μg of RNA was used for cDNA synthesis using a TAKARA PrimeScript RT Reagent Kit. Semiquantitative SYBR green-based RT-PCR was performed using SYBR Premix Ex TaqII (Tli RNaseH Plus). Alpha-*Tubulin* was used as the internal control for normalization. Detailed information for the primers used in the present study is listed in Supplementary Table 1. The presented data are representative of three biological replicates and four technical replicates for each sample. Relative gene expression levels were calculated with the comparative threshold cycle method using a StepOne Plus Real Time PCR System (Applied Biosystems, United States).

### Optimization of Culture Medium for *Phlebopus portentosus*

The medium was optimized based on the results from the genome and transcriptome for suitable carbon sources selection. Stock cultures were grown on complete yeast extract medium (CYM) plates with 2% glucose, 0.2% yeast extracts, 0.2% peptone, 0.1% K_2_HPO_4_, 0.05% MgSO_4_, 0.046% KH_2_PO_4_, and 2% agar. All optimization experiments were carried out in 9-cm-diameter Petri dishes (16 ml/petri dish) with modified CYM medium containing 2% different carbon sources. All the samples cultivated at 30°C in the dark for 30 days. The measurement of hyphal diameters was conducted. The significance of growth rate on different carbon sources was determined using *t-test*.

## Results

### Genome Assembly

Two sequencing libraries (ONT and Illumina) were constructed for the monokaryotic strain for genome assembly. A total of 951,686 long reads (11,348,781,309 bp) were generated using the ONT platform. The N50 and N90 values of the raw data were 18,704 and 6,226 bp, respectively. After filtering the low-quality reads, 792,451 reads (10,603,105,695 bp) were obtained, with N50, N90 and a mean length of reads 18,866, 7,033 and 13,380 bp. The total data provided 323-fold coverage of the genome (Supplementary Table 2). Illumina sequencing yielded a total of 5.56 Gb clean data, and 20,876,612 clean reads (6,208,784,986 bp) for the monokaryotic strain were obtained by RNA-Seq.

An assembly of 32,742,503 bp across 62 scaffolds with a GC content of 48.92% was obtained (Table 1). Based on the results from the BUSCOO pipeline, only 9 of 290 single-copy entries were missing, suggesting > 95.17% genome completeness. Finally, 9,464 putative genes were predicted using a combination of different pipelines (Table 1). A total of 3393, 3175, 5143, 6751, 5552, 9023, and 9231 encoded proteins were identified with homologous sequences in the GO, KEGG, KOG, Pfam, SwissProt, TrEMBL, and Nr databases, respectively (Table 1). The 103 tRNA genes are dispersed in the genome. However, no rRNA genes were identified. Compared with other fungi used in this study, the genome size of *P. portentosus* was the smallest and the number of genes was the least except *Tuber melanosporum* (Table 2).

**TABLE 1:** Statistical information associated with the genome sequenced in this study.

		Value
**Genome size (bp)**	–	**32,742,503**
Scaffolds	–	62
Scaffold N50 (bp)	–	1,263,687
GC (%)	–	48.92
No. of Genes	–	9464
rRNA	–	0
tRNA	–	103
Annotations	GO	3393
	KEGG	3175
	KOG	5143
	Pfam	6751
	Swissprot	5552
	TrEMBL	9023
	Nr	9231
BUSCOs	Complete BUSCOs (C)	276 (95.17%)
	Complete and single-copy BUSCOs (S)	264 (91.03%)
	Complete and duplicated BUSCOs (D)	12 (4.14%)
	Fragmented BUSCOs (F)	5 (1.72%)
	Missing BUSCOs (M)	9 (3.10%)
	Total Lineage BUSCOs	290

**TABLE 2:** The total number of CAZyme families in different fungal genomes.

Ecology inches	Species	Genome size (Mb)	No. of genes	Accession numbers	AA	CBM	CE	GH	GT	PL	Total
	*Phlebopus portentosus*	32,74	9,464	This study	57	19	47	110	62	6	301
Ectomycorrhizal	*Amanita muscaria*	40.70	18,091	JMDV01000001	77	19	61	109	68	4	338
	*Paxillus involutus*	58.30	17,984	JOMD00000000	62	14	64	171	84	9	404
	*Paxillus rubicundulus*	53.01	22,354	JMDR00000000	47	12	39	108	61	6	273
	*Piloderma croceum*	59.33	21,607	JMDN00000000	87	16	71	160	76	7	417
	*Pisolithus microcarpus*	53.03	21,104	JMDM00000000	31	5	30	88	69	4	227
	*Pisolithus tinctorius*	71.01	22,845	JMDO00000000	42	2	38	87	69	3	241
	*Scleroderma citrinum*	56.14	20,993	JMDU00000000	56	4	43	101	63	4	271
	*Suillus luteus*	37.01	18,419	JMSM00000000	57	7	42	131	66	6	309
	*Tuber melanosporum*	124.95	7,496	CABJ00000000	38	8	25	83	58	3	215
	*Laccaria bicolor*	64.88	18,264	ABFE00000000	51	12	49	149	67	8	336
Saprophytic	*Schizophyllum commune*	38.48	13,189	ADMJ00000000	85	22	82	241	77	18	525
	*Lentinula edodes*	41.82	14,889	LDAT00000000	90	40	80	249	78	10	547
	*Volvariella volvacea*	36.45	11,084	AMXZ00000000	121	70	63	215	63	29	561
	*Pleurotus ostreatus*	50.90	12,296	AYUK00000000	139	61	79	233	69	25	606
	*Coprinus cinereus*	38.70	16,862	JAAGWA000000000	132	67	86	192	74	16	567
	*Agaricus bisporus*	30.23	10,448	AEOK00000000	94	31	76	180	57	11	449

*AA, auxiliary activity; CBM, carbohydrate-binding module; CE, carbohydrate esterase; GH, glycoside hydrolase; GT, glycosyl transferase; PL, polysaccharide lyase.*

### Genome Annotations

Gene annotation and functional categorization were performed using EuKaryotic Orthologous Group (KOG), where 5143 genes were redundantly assigned into 25 categories (Table 1 and Supplementary Figure 1). Except for genes related to general function prediction only (R) and unknown function (S), the number of genes involved in posttranslational modification, protein turnover, and chaperones (O); signal transduction mechanisms (T); and translation, ribosomal structure and biogenesis (J) were the highest, reaching 551, 403, and 334, respectively. The genes assigned to defense mechanisms, nuclear structure, cell motility and extracellular structures were the least abundant, with only 37, 28, 6, and 5 identified, respectively.

In addition, 35.85% of total genes had at least one GO annotation. Within the cellular component (CC) category, 4604 genes were assigned to 11 subcategories, the most abundant being “cell” and “cell part” (Table 1 and Supplementary Figure 2). A total of 3035 genes were classified into 13 molecular function (MF) categories, the greatest number of which was catalytic activity, followed by binding. A total of 1,469 predicted genes were assigned to 13 biological process (BP) GO terms, the most heavily represented being metabolic process and cellular process.

A total of 3,175 genes involved in 108 pathways were detected by KEGG annotation. The most enriched pathways included biosynthesis of amino acids (111), ribosomes (111), RNA transport (109), carbon metabolism (98), spliceosome (92), and purine metabolism (84) (Table 1 and Supplementary Figure 3).

### Carbohydrate Metabolism Enzymes Families

A total of 301 CAZyme-coding genes for plant cell wall degradation were detected in the *P. portentosus* genome. These genes were divided into six families, including 57 auxiliary activities (AA) family genes, 19 carbohydrate-binding module (CBM) family genes, 47 carbohydrate esterase (CE) family genes, 110 glycoside hydrolase (GH) family genes, 62 glycosyl transferase (GT) family genes, and 6 polysaccharide lyase (PL) family genes (Table 2). The total number of CAZyme family genes in *P. portentosus* was within the range of that observed for mycorrhizal fungi (Table 2). The average number (303) of CAZyme families detected in the genomes of ectomycorrhizal fungi was much lower (ranging from 215 in *T. melanosporum* to 417 in *Piloderma croceum*) than that of saprophytic fungi (average number 542, ranging from 449 in *A. bisporus* to 606 in *P. ostreatus*) (Table 2). The number of AAs in *P. portentosus* (57 copies) was similar to that observed in *Paxillus involutus* (62 copies). *P. portentosus* had the same number of genes (19) related to CBM families as *Amanita muscaria* (Table 2). The number of genes belonging to GH families predicted in *P. portentosus*, *A. muscaria*, and *P. rubicundulus* was 110, 109, and 108, respectively. No more than 10 copies of PL genes in mycorrhizal fungi were identified. Except for GT families, the number of AA-, CBM-, CE-, GH-, and PL-encoding genes was much lower in ectomycorrhizal fungi than in saprophytic fungi (Table 2).

**FIGURE 1:**
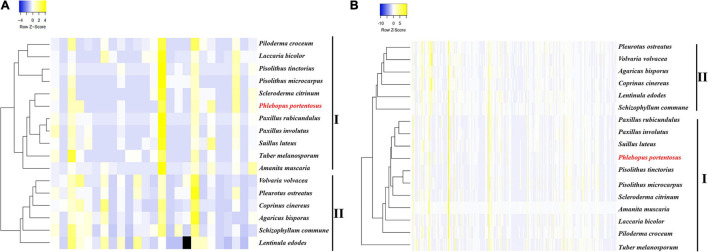
Heatmap clustering of mycorrhizal and saprophytic fungi based on genes related to CAZyme families and lignocellulose decomposition. **(A)** Heatmap clustering based on CAZyme families; **(B)** Heatmap clustering based on genes related to lignocellulose decomposition.

Based on the compositions of CAZyme families, the clustering results revealed two clades that included either mycorrhizal or saprophytic fungi. The pattern of CAZyme genes in *P. portentosus* was similar to that observed in *P. rubicundulus*, *P. involutus*, and *Suillus luteus* (Figure 1). The top five most abundant families in *P. portentosus* were CE10 (36 copies), GH16 (17 copies), AA7 (16 copies), AA1_1 (12 copies), and CBM5 (8 copies). CE10 genes encode esterases, GH16 enzymes act on xyloglucan and chitin and AA7 genes encode gluco- or chito-oligosaccharide oxidases, all of which were abundant in mycorrhizal and saprophytic fungi (Supplementary Table 1). AA1_1 enzymes are multicopper oxidases and represent the most abundant AA family in *P. portentosus* and mycorrhizal fungi assayed in the present study, while AA3_2 enzymes belonging to the glucose-methanol-choline (GMC) oxidoreductase family were the most abundant in saprophytic fungi (Supplementary Table 1). In addition, the families GH6 and GH7, which are involved in attacking crystalline cellulose, were present in all saprophytic fungi assayed in the present study and were absent in *P. portentosus* and other ectomycorrhizal fungi (Supplementary Table 1). The families GH13, GH15, GH27, and GH71 were enriched in *P. portentosus*, of which the copy numbers of the starch degradation-associated GH13, GH27, and GH71 families were higher than those observed in other mycorrhizal fungi (Supplementary Table 1).

### Genes Involved in Lignocellulose Decomposition

Low copy numbers of genes were identified belonging to the cellulase, hemicellulase, pectinase, and lignin oxidase families, which directly act on lignocellulose and are listed in Table 3. Hierarchical clustering using the average-linkage method based on the composition of lignocellulases also resulted in the identification of two clades, revealing the different compositions of lignocellulases between ectomycorrhizal and saprophytic fungi (Figure 1B).

**TABLE 3:** The distribution of genes related to lignocellulose degradation in *P. portentosus* and other fungi.

	Enzyme	EC	Saprophytic						Ectomycorrhizal										
		
			SC	LE	PO	CC	VV	AB	PP	Lb	AM	PI	PR	PC	PM	PT	SCC	SL	TM
Cellulase	Endo-beta-1,4-glucanase	EC:3.2.1.4	4	5	9	4	4	4	1	2	1	6	3	2	1	1	2	3	2
	1,4-β-cellobiosidase	EC:3.2.1.91	1	0	3	5	5	1	0	0	0	0	0	0	0	0	0	0	0
	β-glucosidase	EC:3.2.1.21	12	11	12	9	13	5	3	2	3	6	4	11	3	2	5	6	6
	Total		17	16	24	18	22	10	4	4	4	12	7	13	4	3	7	9	8
Hemicellulase	Endo-1,4-betaxylanase	EC:3.2.1.8	6	0	5	12	18	4	3	0	1	1	1	0	0	0	0	1	1
	β-xylosidase	EC:3.2.1.37	4	5	5	4	3	5	0	0	1	3	1	1	0	0	0	4	0
	α-glucuronidase	EC:3.2.1.131	2	1	1	1	3	2	0	0	0	0	0	0	0	0	0	0	0
	Acetylxylan esterase	EC:3.1.1.72	9	1	7	18	10	7	0	6	3	0	0	4	0	0	0	0	0
	Feruloyl esterase	EC:3.1.1.73	1	0	0	0	0	0	0	2	1	0	0	0	0	0	0	0	0
	α-L-arabinofuranosidases	EC:3.2.1.55	7	3	5	4	6	2	1	0	0	1	1	2	1	2	0	2	0
	Total	–	29	10	23	39	40	20	5	8	6	5	3	7	1	2	0	7	1
Pectinase	Pectin lyase	EC:4.2.2.10	0	0	0	0	0	0	0	0	0	0	0	0	0	0	0	0	1
	Pectate lyase	EC:4.2.2.2	10	3	11	3	13	3	0	0	0	0	0	1	0	0	0	0	1
	Pectinesterase	EC:3.1.1.11	2	3	2	0	3	2	0	3	0	0	0	2	0	0	0	2	0
	Polygalacturonase	EC:3.2.1.15	1	5	1	0	0	1	0	3	0	2	1	3	1	1	2	1	1
	Total	–	13	11	14	3	16	6	0	6	0	2	1	6	1	1	2	3	3
Lignin Oxidase	Multicopper oxidase	1.10.3.2	4	13	12	17	11	9	6	14	19	17	16	18	9	12	11	18	5
	Lignin peroxidase	1.11.1.14	0	0	0	0	0	0	0	0	0	0	0	0	1	0	0	0	0
	Manganese peroxidase	1.11.1.13	0	8	5	1	2	2	1	1	0	0	0	0	1	1	1	0	0
	Other peroxidase	1.11.1.16	2	1	5	2	8	3	0	1	1	0	0	0	1	0	0	0	0
	Total	-	6	22	22	20	21	14	7	16	20	17	16	18	12	13	12	18	5
	Aryl-alcohol oxidase	1.1.3.7	7	13	35	21	25	16	3	4	5	5	5	16	3	4	4	4	3
Lignin Degrading Auxiliary Enzyme	Glucose oxidase	1.1.3.4	7	2	0	2	0	0	1	1	0	0	0	4	0	0	1	1	0
	Alcohol oxidase	1.1.3.13	4	4	4	2	5	4	4	2	5	3	2	2	0	0	1	11	1
	Pyranose oxidase	1.1.3.10	1	1	0	0	0	0	0	0	0	0	0	0	0	0	0	0	0
	Vanillyl-alcohol oxidase	1.1.3.38	0	2	1	0	1	1	0	1	0	0	0	0	0	0	0	0	0
	Glyoxal oxidase	1.1.3.-	2	4	12	5	3	0	4	6	3	4	3	5	4	4	6	4	1
	Galactose oxidase	1.1.3.9	0	0	0	0	0	0	0	2	0	0	0	0	0	0	0	0	0
	Benzoquinone reductase	1.6.5.6	4	2	2	3	2	4	2	2	8	2	2	2	1	2	3	2	1
	Total	–	18	28	54	33	36	25	12	18	21	14	12	29	8	10	15	22	6

*AS, *Agaricus bisporus*; AM, *Amanita muscaria*; CC, *Coprinus cinereus*; LB, *Laccaria bicolor*; LE, *Lentinula edodes*; PI, *Paxillus involutus*; PR, *Paxillus rubicundulus*; PP, *Phlebopus portentosus*; PC, *Piloderma croceum*; PM, *Pisolithus microcarpus*; PT, *Pisolithus tinctorius*; PO, *Pleurotus ostreatus*; SC, *Schizophyllum commune*; SCC, *Scleroderma citrinum*; SL, *Suillus luteus*; TM, *Tuber melanosporum*; VV, *Volvariella volvacea.**

Four cellulase genes were identified in the *P. portentosus* genome, including 1 endo-beta-1,4-glucanase and 3 β-glucosidases. However, 1,4-β-cellobiosidase, which specifically hydrolyzes 1,4-beta-D-glucosidic linkages, was absent in *P. portentosus* and other ectomycorrhizal fungi, suggesting that *P. portentosus* may also have a low capacity to degrade celluloses. A low copy number of genes related to hemicellulases was observed in *P. portentosus*, with only five hemicellulases (no higher than seven in other ectomycorrhizal fungi) detected compared to saprophytic fungi (ranging from 10 to 29). In particular, no hemicellulases were detected in *S. citrinum*. α-Glucuronidases were absent in all ectomycorrhizal fungi evaluated in the present study, whereas they were present in all assayed saprophytic fungi. In *P. port*entosus, no pectinase genes were identified, as was observed in *A. muscaria*. The number of pectinases in ectomycorrhizal fungi ranged from 0 to 6. In saprophytic fungi, the copy number of pectinases varied significantly in different species, e.g., 3 present in *Coprinus cinereus* and 16 present in *Volvariella volvacea*. In addition, pectate lyases, which were detected in all saprophytic fungi, were absent in ectomycorrhizal fungi.

Six multicopper oxidases and one manganese peroxidase were identified in *P. portentosus*, fewer than was observed in other ectomycorrhizal fungi belonging to Basidiomycota. The number of multicopper oxidases encoded by these fungi was not in agreement with their associated niches and detected in all the fungi. The same pattern of auxiliary enzymes (e.g., aryl-alcohol oxidase, glyoxal oxidase, and benzoquinone reductase) was observed in all the fungi assayed in the present study. Pyranose oxidase, vanillyl-alcohol oxidase and galactose oxidase were not detected in any of the ectomycorrhizal fungi, including *P. portentosus*, except for *L. bicolor*.

### Gene Expression Profiles in Different Stages

To determine the gene expression patterns during *P. portentosus* development, 9 samples from 3 growth stages were used for RNA-Seq. The average number of raw reads was 51,592,958, ranging from 47,344,648 in sample S3 to 56,606,312 in sample F3 (Supplementary Table 3). After filtering, the average numbers of clean reads and total bases was 51,161,401 and 7,631,017,892, ranging from 46,876,694 and 6,987,544,020 in sample S3 to 56,203,088 and 8,367,222,043 in sample F3, respectively (Supplementary Table 3). More than 87.62% of the reads within each replicate could be mapped to the reference genome (Supplementary Table 3).

Using an FPKM cutoff value of 1, 8,556, 8,628 and 8,673 genes expressed in stages S, P, and F accounted for 90.40, 91.17, and 91.64% of the total predicted genes, respectively (Supplementary Figure 4A). A total of 8,316 common genes were expressed in all three stages. According to the FPKM values in different stages (FPKM < 10, 10–100, and >100), three gene categories were determined (Supplementary Figure 4B). The majority of genes in all the samples showed moderate expression, with FPKM values ranging from 10 to 100 (Supplementary Figure 4B). The F stage had the greatest number of expressed genes, while the S stage had the fewest. However, the F stage had the fewest highly expressed genes, while the P stage had the most. To determine the correlations among different stages, the nine samples were clustered into three groups based on principal component analysis, which revealed different patterns of gene expression across all stages (Supplementary Figure 5).

### Differentially Expressed Genes Across Different Developmental Stages

**FIGURE 2:**
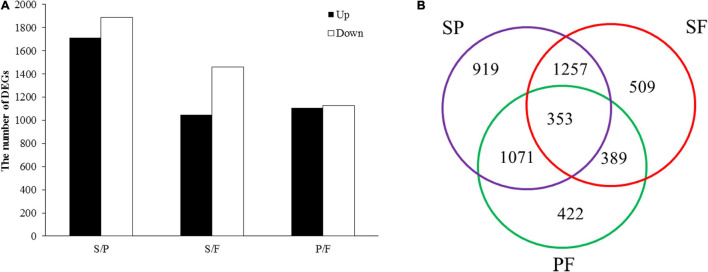
Analysis of DEGs between different developmental stages. **(A)** DEG distribution between two analyzed samples. The number of differentially expressed genes is indicated at the top of the histograms. **(B)** Venn diagrams comparing shared DEGs between the adjacent growth stages.

Based on FPKM values (>1), a corrected *P*-value of 0.05 and log2 (fold change) of 1 were set as the threshold for significantly different gene expression. A total of 4,921 DEGs were identified in comparisons between the three groups (S vs. P, S vs. F, and P vs. F) (Figure 2). The number of DEGs between the S and P groups was the highest (3600), followed by the S vs. F (2508) and P vs. F (2235) comparisons (Figure 2B). There were 353 common DEGs in all three growth stage comparisons, which were enriched in the carbohydrate metabolism and proteasome categories (Figure 2B). The most unique DEGs (919) were observed between the S and P groups compared to the S vs. F (509) and P vs. F (422) comparisons (Figure 2A). The numbers of downregulated genes in the S vs. P, S vs. F and P vs. F comparisons were higher than those of upregulated genes (Figure 2A).

### Functional Classification of Differentially Expressed Genes

**FIGURE 3:**
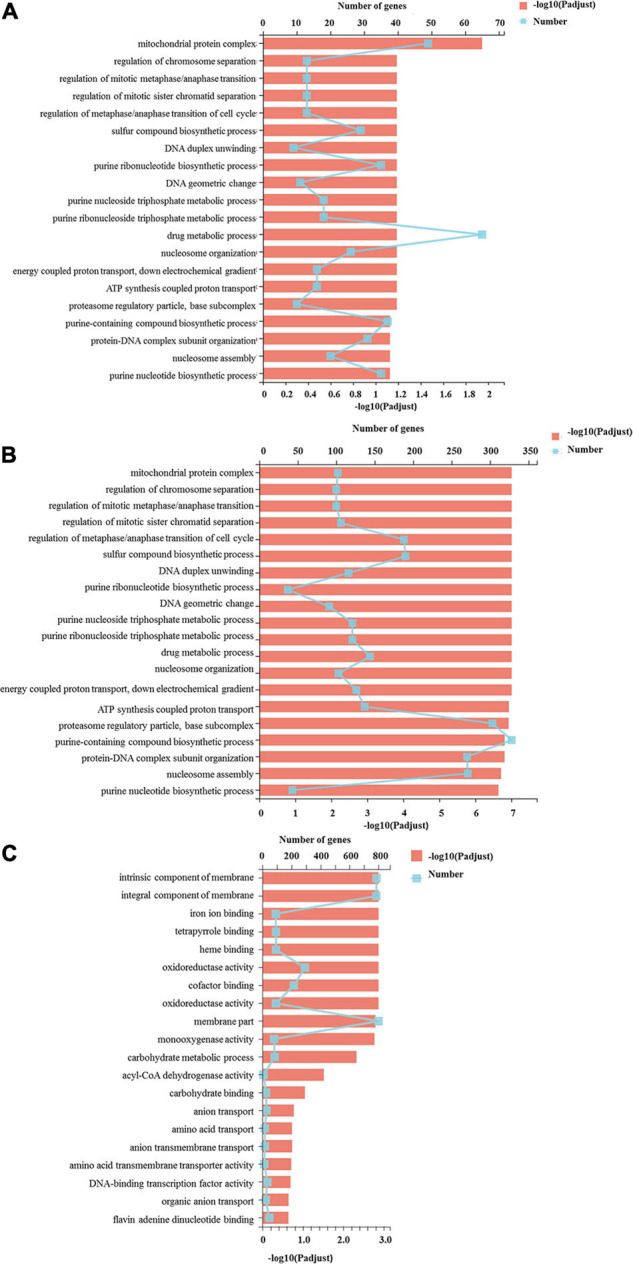
GO functional classification of differentially expressed genes. The *Y* axis represents the GO terms, the upper *X* axis represents the number of DEGs assigned to GO terms, the basal *X* axis represents the *p*-adjusted value, the broken lines represent the number of DEGs in each GO term, and the bars represent the *p*-adjusted value. **(A)** The DEGs assigned to GO terms in the S vs. P comparison; **(B)** indicated the DEGs assigned to GO terms in the P vs. F comparison; **(C)** the DEGs assigned to GO terms in the S vs. F comparison.

All the DEGs were assigned into three different functional GO categories and 41 subcategories, including biological process (BP, 15 subcategories), cellular component (CC, 11 subcategories) and molecular function (MF, 15 subcategories) (Figure 3). The most enriched DEGs were assigned to catalytic activity (GO: 0003824) in all three comparisons. The DEGs in the S vs. P comparison were significantly enriched in drug metabolic process, mitochondrial protein complex and purine-containing compound biosynthetic process. Functional categories for the P vs. F comparison were significantly enriched for gene families associated with cytoplasmic part, organic substance biosynthetic process, cellular biosynthetic process and organic substance biosynthetic process.

**FIGURE 4:**
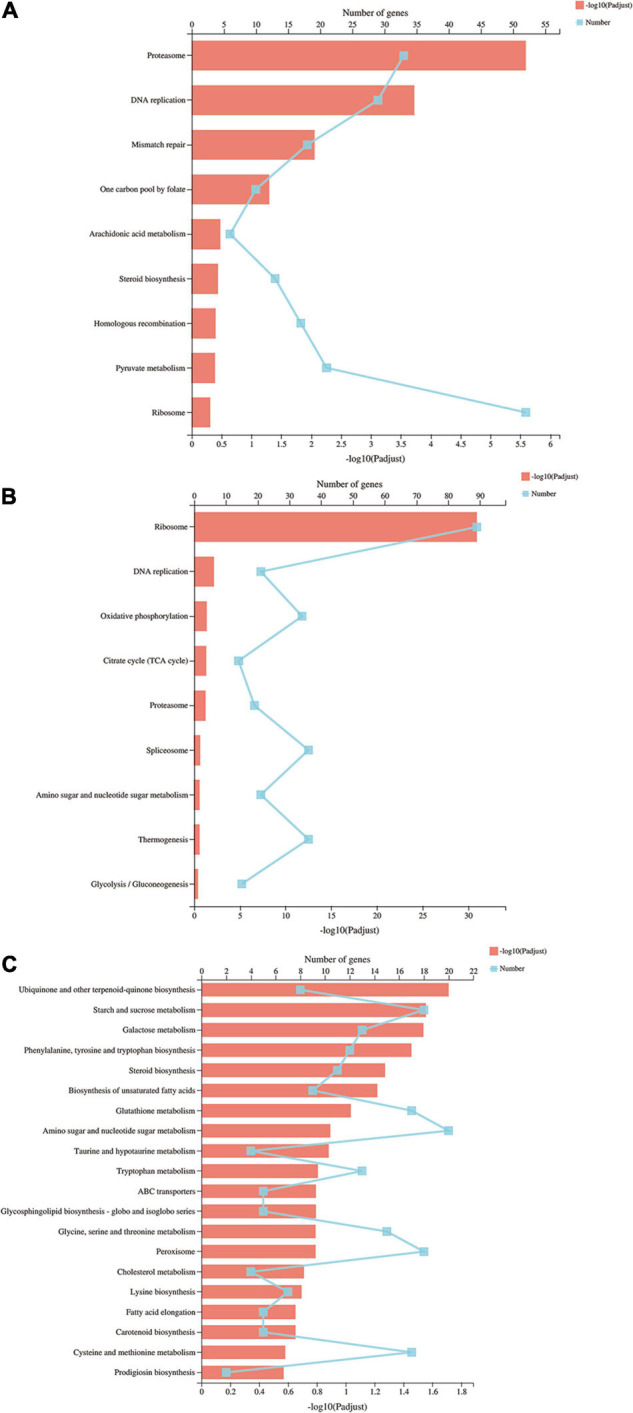
KEGG pathways of differentially expressed genes. The *Y* axis represents the KEGG pathways, the upper *X* axis represents the number of DEGs, the basal *X* axis represents the *p*-adjusted value, the broken lines represent the number of DEGs in each KEGG pathway, and the bars represent the *p*-adjusted value. **(A)** KEGG pathways of differentially expressed genes in the S vs. P comparison; **(B)** KEGG pathways of differentially expressed genes in the P vs. F comparison; **(C)** KEGG pathways of differentially expressed genes in the S vs. F comparison.

To further understand the molecular and biological functions of the DEGs, they were mapped to the KEGG database (Figure 4). Pathway enrichment analysis revealed that proteasome and DNA replication were the most enriched in the S vs. P comparison. The ribosome pathway was the most significant pathway in the P vs. F comparison, with a *P*-value of nearly 0, following by DNA replication, oxidative phosphorylation, citrate cycle and peroxisome. Amino sugar and nucleotide sugar metabolism, peroxisome and cysteine and methionine metabolism were the most enriched in the S vs. F comparison.

### Gene Coexpression Network Construction

**FIGURE 5:**
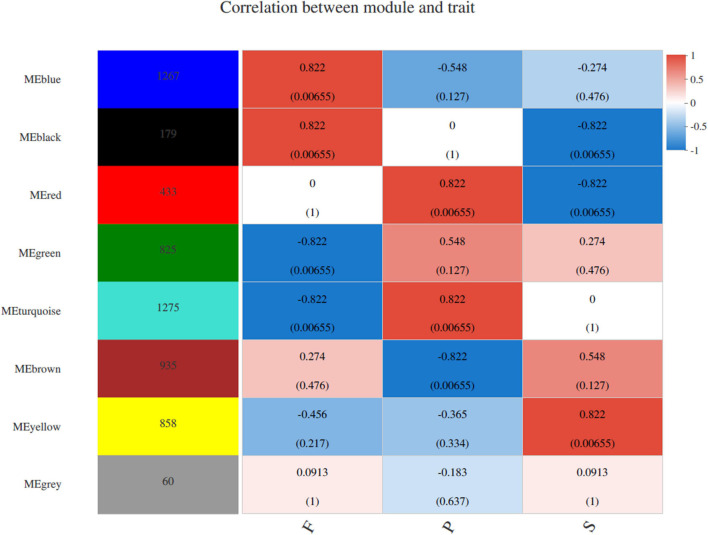
WGCNA of genes in different developmental stages in *P. portentosus*. Module-trait weighted correlations and corresponding *P*-values for the identified gene module and their developmental stages. The label color on the right represents the strength of correlation, from 1 (red) to –1 (blue). Each column corresponds to a developmental stage. The eight modules and the number of module member genes are shown on the left panel. The boxes with different color indicated the significant correlation between the module eigengene and the stage.

WGCNA was conducted to uncover the coexpression profiles in successive developmental stages (Figure 5). Eight different modules were identified with high correlation coefficients according to the similarity of expression patterns (Supplementary Tables 5–12). To identify key genes specific to the S stage, the yellow module was analyzed and included a total of 858 genes. Amino sugar and nucleotide sugar metabolism, pyrimidine metabolism, biosynthesis of amino acids, purine metabolism and starch and sucrose metabolism were the most enriched pathways (Supplementary Table 5). Regarding the amino acids biosynthesis pathways, those involved in phenylalanine, tyrosine and tryptophan biosynthesis, histidine metabolism and glycine, serine and threonine metabolism were the most abundant (Supplementary Table 5). The turquoise and red modules (harboring 1,275 and 433 identified genes, respectively) were specific to the P stage, the number of genes in which was higher than was observed in other two stages. In this stage, RNA transport, cell cycle (DNA replication and meiosis), proteasome and spliceosome were the most enriched pathways (Supplementary Tables 6,7). The blue (63 genes) and black (63 genes) modules were highly associated with the F stage. Biosynthesis of amino acids, glutathione metabolism, peroxisome, glycerophospholipid metabolism and MAPK signaling pathway were the most enriched in this stage (Supplementary Tables 8,9).

### Differential Expression of Carbohydrate Metabolism Enzymes Families and Lignocellulose Decomposition-Related Genes

Of the 301 CAZyme genes predicted from the genome, 277, 286 and 288 genes were expressed in the S, P, and F stages, respectively (Supplementary Table 4). The most highly expressed CAZyme genes were different in the three stages (Supplementary Table 4). In the S stage, the top five families with the highest FPKM values were CBM13 (1872.75), GH16 (1230.55), GH128 (1168.78), CBM13 (923.36) and AA5_1 (532.96). In the P stage, AA2 (1391.67), GT4 (992.50), GH128 (867.38), AA3_2 (298.65) and CBM50 (288.51) were more highly expressed than other families. The families GH16 (6346.44), GH18 (1097.48), GH128 (1003.62), GT4 (969.11), and GT2_Glyco_trans_2_3 (660.98) were overrepresented in stage F. The high expression of the glycoside hydrolase families GH16, GH18 and GH128 in all three stages indicated that *P. portentosus* has a high potential for starch or xylan degradation.

A total of 106 and 78 DEGs annotated as CAZyme families were detected in the S vs. P and P vs. F comparisons, respectively (Supplementary Table 4). In the S vs. P comparison, out of the 106 identified DEGs, 46 were upregulated and 60 were downregulated. Compared to the S stage, CBM13, GH16, AA5_1, GH18, AA6, AA3_2, GH17, GH5_30 and GT2_Chitin_synth_1 were significantly downregulated and AA2, GT4 and GH71 were upregulated in the P stage. In the P vs. F comparison, 52 DEGs were upregulated, including GH16, GH18, GT2_Glyco_trans_2_3, GH13_1 and GH31, while 26 DEGs were downregulated, including AA2, GH16, CE10, GH16, and CE4.

### Differential Expression of Genes Involved in Lignocellulose Decomposition

**FIGURE 6:**
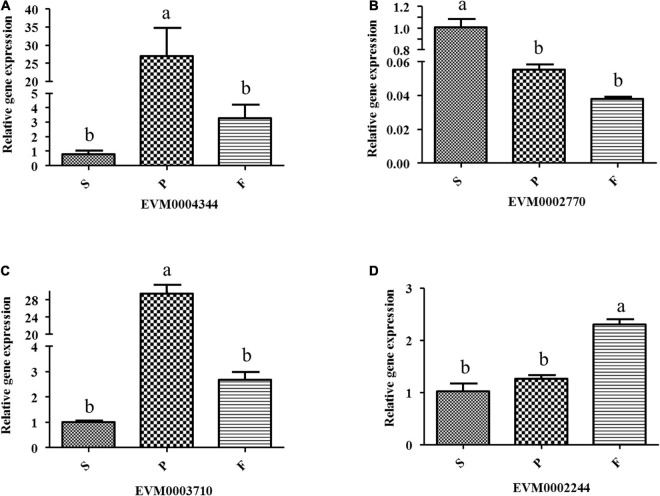
Relative expression of some lignin oxidases and cellulases in different developmental stages of *P. portentosus* using RT-PCR. **(A)** Manganese peroxidase; **(B)** multicopper oxidase; **(C)** endo-beta-1,4-glucanase; **(D)** β-glucosidase. Different lowercase letters above bars indicate a significant difference according to the Duncan’s multiple range test (*p* < 0.05).

Twenty-four genes involved in lignocellulose decomposition were predicted from the *P. portentosus* reference genome, of which 15 genes were differentially expressed in the different developmental stages (Table 4). The most highly expressed genes involved in lignocellulose decomposition in all 3 stages included lignin oxidases (manganese peroxidase) and lignin-degrading auxiliary enzymes (alcohol oxidase, benzoquinone reductase), suggesting that *P. portentosus* has the potential to degrade lignin (Table 4). However, endo-beta-1,4-glucanase, which acts on cellulose, was expressed at low levels. Compared to that observed in the S stage, the expression of manganese peroxidase (EVM0004344) in the P stage was significantly upregulated (293.84 vs. 1391.67, respectively, a nearly fivefold increase) (Table 4), the expression of which subsequently decreased in the F stage. The RT-PCR results confirmed the observed expression profiles (Figure 6A). Multicopper oxidase laccases (EVM0002770, EVM0008799, and EVM0004198) were expressed at the highest levels in the S stage (Table 4 and Figure 6B). The lignin-degrading auxiliary enzymes glyoxal oxidase (EVM0006667), benzoquinone reductase (EVM0006980 and EVM0009178) and glucose oxidase (EVM0003870) were more highly expressed in the P stage than in the S and F stages (Table 4). The expression of aryl-alcohol oxidase (EVM0008750) gradually increased with the development of fruiting bodies, peaking in the F stage (Table 4). Endo-beta-1,4-glucanase expression was lower in the S (11.68) and F (23.71) stages than in the P stage (46.76) (Table 4). The expression of β-glucosidase (EVM0002244) increased from stages P (29.79) to F (105.70) (Table 4 and Figure 6D). Endo-1,4-betaxylanase (EVM0003710), which acts on hemicellulose, was expressed at higher levels in the F stage than in the P and S stages (Table 4 and Figure 6C).

**TABLE 4:** FPKM of DEGs annotated as lignocellulose degradation enzymes.

Class	Gene_id	Enzymes	CAZyme	FPKM		
			**Families**	**S**	**P**	**F**
Cellulase	EVM0003710	Endo-beta-1,4-glucanase	GH9	11.68 ± 1.74*b*	46.76 ± 19.02*a*	23.71 ± 8.09*ab*
	EVM0002244	β-glucosidase	GH3	25.15 ± 4.58*b*	29.79 ± 13.71*b*	105.70 ± 29.30*a*
Hemicellulase	EVM0005879	Endo-1,4-betaxylanase	CBM5	5.10 ± 0.25*ab*	3.63 ± 0.59*b*	9.77 ± 1.86*a*
Pectinase	EVM0009029	Polygalacturonase	GH43_30	51.62 ± 18.11*a*	16.89 ± 12.02*a*	1.67 ± 0.49*b*
Lignin	EVM0004344	Manganese peroxidase	AA2	293.84 ± 68.16*b*	1391.67 ± 320.55*a*	473.57 ± 172.23*b*
Oxidase	EVM0002770	Multicopper oxidase	AA1_1	118.32 ± 40.67*a*	4.22 ± 2.22*b*	6.99 ± 0.55*b*
	EVM0008799	Multicopper oxidase	AA1_1	15.60 ± 3.39*a*	1.51 ± 0.36*c*	5.44 ± 0.12*b*
	EVM0004198	Multicopper oxidase	AA1_1	2.18 ± 0.44*ab*	1.94 ± 1.03*b*	4.95 ± 0.24*a*
Lignin	EVM0003870	Glucose oxidase	AA3_2	267.35 ± 82.40*a*	43.04 ± 19.44*b*	33.42 ± 25.87*b*
Degrading	EVM0006667	Glyoxal oxidase	AA5_1	532.96 ± 102.57*a*	140.47 ± 3.04*b*	310.52 ± 88.23*b*
Auxiliary	EVM0006812	Glyoxal oxidase	AA5_1	1.72 ± 0.46*b*	3.10 ± 0.62*ab*	4.06 ± 0.38*a*
Enzyme	EVM0006980	Benzoquinone reductase	AA6	447.79 ± 91.82*a*	43.71 ± 12.46*b*	21.42 ± 7.32*b*
	EVM0009178	Benzoquinone reductase	AA6	187.93 ± 28.24*a*	103.21 ± 23.98*ab*	60.87 ± 24.63*b*
	EVM0000957	Alcohol oxidase	AA3_3	110.40 ± 26.76*a*	46.95 ± 11.80*b*	79.24 ± 53.18*ab*
	EVM0008750	Aryl-alcohol oxidase	AA3_2	9.36 ± 0.74*c*	33.56 ± 6.94*b*	68.21 ± 4.58*a*

*Different lowercase letters above bars indicate a significant difference according to the Duncan’s multiple range test (*p* < 0.05).*

### Optimization of Culture Medium

**FIGURE 7:**
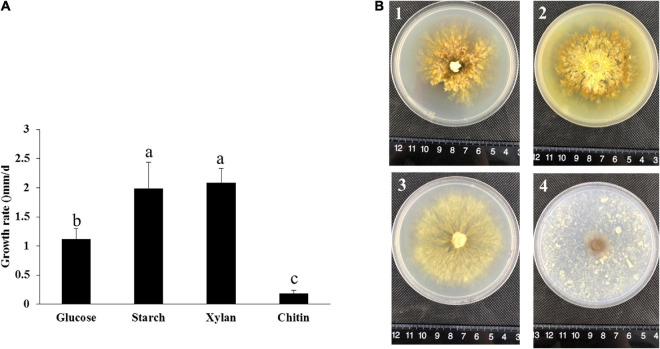
The growth rate and morphology of *P. portentosus* cultured on different carbon sources. **(A)** Growth rate; **(B)** morphology of *P. portentosus* on different carbon sources after 30 days of cultivation, **(B1)** glucose, **(B2)** starch, **(B3)** xylan, **(B4)** chitin. Different lowercase letters above bars indicate a significant difference according to the Duncan’s multiple range test (*p* < 0.05).

From the genome and transcriptome results, *P. portentosus* exhibited a limited ability to degrade lignocellulose. The gene families acting on starch, xylan and chitin were most abundant in the genome and showed high expression in all the stages. Unique carbon sources (starch, xylan, and chitin) were further used to screen the most suitable culture medium, glucose as a control. By measuring the growth diameter of mycelia after 30 days of cultivation, the growth rate of *P. portentosus* on starch and xylan was much faster than that on glucose and chitin (Figure 7A). In addition to the growth rate, the morphology of the colonies also differed. Aerial mycelia of *P. portentosus* were much thicker, and pigmentation was stronger on glucose and starch than on xylan (Figure 7B). However, the integrity of mycelial edges were much better on xylan than on glucose and starch (Figure 7B). Thus, xylan and starch may be more suitable carbon sources for *P. portentosus* than chitin.

## Discussion

As the first ectomycorrhizal fungus in the order Boletaceae to have been industrially cultivated at a large scale, *P. portentosus* has attracted extensive attention worldwide (Zhang et al., 2017). However, the molecular mechanisms associated with the ability of *P. portentosus* to promote plant cell wall degradation and fruiting body development remain ambiguous. Genomics and transcriptomics provide an unprecedented means of elucidating the developmental and metabolic biological aspects of mushrooms. In the present study, genome and transcriptome sequencing was conducted to identify CAZyme family genes and DEGs in different developmental stages of *P. portentosus*. The results revealed the presence of no or few copies of carbohydrate metabolism enzymes (CAZymes) acting on cellulose, xylan, pectin and lignin in *P. portentosus*, an ectomycorrhizal fungus (Kohler et al., 2015). Some conserved pathways related to the proteasome, DNA replication, ribosome pathway and amino acid metabolism processes were correlated with the development of fruiting bodies.

Ectomycorrhizal fungi are considered mutualists that trade host photoassimilates for nutrients (Clavel et al., 2021). ECM fungi have been reported to have evolved from saprotrophic fungi and exhibit an extensive loss of enzymatic repertoires for lignocellulose decomposition during the transition to mycorrhizal habits (Kohler et al., 2015). The use of coculture and isotopic methods revealed that *P. portentosus* can form ectomycorrhizal symbioses with plants (Kumla et al., 2016). However, this fungus can be artificially cultivated *in vitro*, and an improved understanding of the repertoires of PCWDEs in *P. portentosus* is needed to elucidate the mechanisms associated with nutrient acquisition *in vitro*.

The composition of lignocellulose-degrading systems harbored by *P. portentosus* revealed ECM characteristics. Members of the families GH6 and GH7, which degrade crystalline cellulose (Baldrian and Valáková, 2008; Floudas et al., 2012), are absent in *P. portentosus* and other ECM fungi assayed in the present study except *Piloderma croceum* (1 GH7 copy). In addition to GH6 and GH7, fewer AA9 lytic polysaccharide monooxygenases (2–5 copies, LPMOs) were present in all the genomes of ectomycorrhizal fungi assayed in the present study than in white-rot or straw-rot fungi (11–34). These results are in agreement with reports of ECM fungi belonging to Boletales, which have lost nearly all copies of GH6 and GH7 genes and have no more than five copies of AA9 LPMOs (Kohler et al., 2015). These results also suggested that ECM fungi, including *P. portentosus*, have a limited capacity to degrade cellulose. In addition, no genes related to pectin degradation were detected in *P. portentosus* and *A. muscaria*. The lack of pectinases in ECM fungi has also been demonstrated using omics analyses (Kohler et al., 2015). Genes encoding enzymes mediating the degradation of pectin were shown to be reduced or completely lost among EM *Amanita* (Jaqueline et al., 2018). Each species used in the study had a different repertoire of multicopper oxidase (MCO) genes, ranging from 4 to 13 in saprophytic fungi and from 5 to 19 in ECM fungi (Bödeker et al., 2009; Kohler et al., 2015; Shah et al., 2016). Six laccases (MCO) were identified in the *P. portentosus* genome, higher than that observed for *S. commune* but lower than other ECM fungi (except *T. melanosporum*). In addition to the low copy number of lignocellulose-degrading genes observed in the *P. portentosus* genome, the RNA-seq analysis of the different stages showed the low expression of these genes, with measured FPKM values of half of these lignocellulose-degrading genes less than 10. These results were also in agreement with the characteristics of ectomycorrhizal fungi revealed by omics analyses in other studies, and ectomycorrhizal fungi have low potential to degrade lignocellulose (Martin et al., 2016; Shah et al., 2016; Miyauchi et al., 2020).

Although *P. portentosus* was shown to have few copies and low expression of lignocellulose enzymes, the expression of these genes was also dependent on the stage of mushroom development. The expression of endo-beta-1,4-glucanase, β-glucosidase and endo-1,4-betaxylanase was highest in the P or F stages, which may suggest that cellulase and hemicellulase genes are potentially correlated with fruiting body formation and maturation. These results are in agreement with those obtained for some white-rot or straw-rot mushrooms, e.g., *A. bisporus*, *P. ostreatus*, *L. edodes*, *L. tigrinus*, and *G. lucidum* (Shoji and Royse, 2001; Lechner and Papinutti, 2006; Elisashvili et al., 2008; Song et al., 2018; Wu et al., 2018; Zhou et al., 2018). The activities of multicopper oxidases (laccases) and most lignin-degrading auxiliary enzymes reached their maximum levels in the M stage, similar to other mushrooms (Shoji and Royse, 2001; Lechner and Papinutti, 2006; Elisashvili et al., 2008), suggesting the potential to degrade lignin during the mycelial growth phase. Manganese peroxidase activity was reported to be high during the colonization stage and decrease during the first primordia and fruiting body formation stages in some saprophytic mushrooms (Velázquez-Cedeño et al., 2002; Elisashvili et al., 2008). However, the relative expression of manganese peroxidases in *P. portentosus* was inconsistent with previous reports, reaching the highest levels in the primordia stage and then decreasing in the fruiting body formation stage. The expression patterns of these enzymes in *P. portentosus* need to be further studied.

Some conserved and specific pathways have been shown to be enriched in the different stages of mushroom development (Wang et al., 2013, 2018; Zhang et al., 2015; Song et al., 2018; Xie et al., 2018; Zhou et al., 2018; Hao et al., 2019; Wei et al., 2019; Tang et al., 2020). Starch and sucrose metabolism pathways were enriched in the mycelial stage in *P. portentosus*, which were also specific to the hyphal stage of Chinese *Cordyceps* (Li et al., 2019). Starch and sucrose may be important carbon sources for the growth of mycelia. Cultivation using starch and xylan as substrates demonstrated that metabolism plays important roles in acquiring nutrient acquisition in *P. portentosus*. Some ectomycorrhizal fungi belonged to *Amanita*, *Boletus*, *Laccaria*, and *Tricholoma* revealed the ability to utilize starch as a growth substrate (Ohta, 1997; Kusuda et al., 2006). *Tuber maculatum* mycelium utilized xylan as energy source during the growth cycle (Bedade et al., 2017). Both DNA replication and proteasome were enriched in P and F stages in *P. portentosus*, which were detected to be associated with development and maturation in other mushrooms (Masato et al., 2010; Rodenburg et al., 2018; Song et al., 2018; Wang et al., 2018). DNA replication is important for premeiotic replication, karyogamy, and meiosis during maturation in mushrooms, e.g., *L. edodes* (Song et al., 2018; Wang et al., 2018) and *Botrytis cinerea* (Rodenburg et al., 2018). The proteasome is presumably linked with fruiting body development in basidiomycetes by degrading unnecessary proteins through the ubiquitin-proteasome pathway (Masato et al., 2010). Furthermore, peroxisome, ribosome, oxidative phosphorylation, citrate cycle and MAPK signaling pathways were enriched in the stage of F in *P. portentosus.* Ribosome pathways were increased significantly during the fruiting body growth stage in *Ophiocordyceps sinensis* (Zhang et al., 2021), *F. velutipes* (Liu et al., 2014), *Auricularia polytricha* (Zhou et al., 2014), and *Agaricus blazei* (Lu et al., 2020) to provide proteins for fruiting body formation or carry out some other functions, e.g., DNA repair, development, and cell division. In fungi, the activity of peroxisomes was required for sexual development (Peraza-Reyes and Berteaux-Lecellier, 2013). Oxidative phosphorylation and citrate cycle were the key metabolic pathways for the growth and development of *Clavariadelphus pistillaris* (Tang et al., 2019). Higher expression of MAPK genes involved in MAPK signaling pathways is required for fruiting in some fungi, which plays an important role in promoting sexual reproduction, regulating the polar growth of mycelia and rebuilding of the cell wall in the mature fruiting body (Pöggeler et al., 2006; Zhang et al., 2015; Li et al., 2019).

## Conclusion

Our results revealed fewer cell wall-degrading enzymes encoded in the genome of *P. portentosus* than that observed in some saprophytic mushrooms with specific ectomycorrhizal niches. The low expression of plant cell wall-degrading genes confirmed the limited ability of *P. portentosus* to degrade lignocellulose. In addition, some conserved and specific pathways were enriched in the different developmental stages of *P. portentosus*, e.g., starch and sucrose metabolism enriched in the mycelial stage and DNA replication, proteasome and MAPK signaling pathways enriched in the fruiting body stage. The results of the present study advance our understanding of the molecular mechanisms of *P. portentosus* lignocellulose degradation and fruiting body development.

## Data Availability Statement

The datasets presented in this study can be found in online repositories. The names of the repository/repositories and accession number(s) can be found below: https://www.ncbi.nlm.nih.gov/, PRJNA743691 (SRR15039576–SRR15039584). https://www.ncbi.nlm.nih.gov/, JAHRGP000000000.

## Author Contributions

R-HY, D-PB, K-PJ, S-ZL, and QT contributed to the conception and design of the study. J-NW, YL, S-ZL, YC, and G-YJ prepared the samples. R-HY and K-PJ performed the analysis and wrote the manuscript draft. All authors approved the final version of the manuscript.

## Conflict of Interest

K-PJ, YC, S-ZL, and G-YJ are employed by Hongzhen Agricultural Science and Technology Co. Ltd. The remaining authors declare that the research was conducted in the absence of any commercial or financial relationships that could be construed as a potential conflict of interest.

## Publisher’s Note

All claims expressed in this article are solely those of the authors and do not necessarily represent those of their affiliated organizations, or those of the publisher, the editors and the reviewers. Any product that may be evaluated in this article, or claim that may be made by its manufacturer, is not guaranteed or endorsed by the publisher.

## Footnotes

1
http://genome.jgi.doe.gov/31_SAP
