# Supplementary material for: Whole-Genome and Transcriptome Sequencing of Phlebopus portentosus Reveals Its Associated Ectomycorrhizal Niche and Conserved Pathways Involved in Fruiting Body Development
Source: Front Microbiol. 2021 Sep 29;12:732458. doi: 10.3389/fmicb.2021.732458 (PMC8511702; doi:10.3389/fmicb.2021.732458)
Supplement: Supplementary file 1 [file Data_Sheet_1.docx]

Table S1. The detailed information of the primers used for RT-PCR in this study

| Primers | Forward | Reverse |
| --- | --- | --- |
| Alpha-*Tubulin* | TGCCTGCAAGGTTTCCAGAT | TGTATGGTTCGACGACGGTG |
| EVM0004344 | AAATCGTAGCCCTTTGCGGA | TCGTTGGTGAACGTGGTAGG |
| EVM0003710 | CGCTGCATATACGGAGGGTT | ACCCCACCAGCCAACTAATG |
| EVM0002770 | ATTGCTCCGGGACATTCGTT | AAGTAGGGATCCTGGGGGTC |
| EVM0002244 | AACAGCATGACCCCATCGAG | CGAAGGTGCAGTCATGGTCT |

Table S2 the raw data for the genome sequencing

|  | Raw data | Clean data |
| --- | --- | --- |
| No. of the sequences | 951,686 | 792,451 |
| Total base | 11,348,781,309 | 10,603,105,695 |
| N50 | 18,704 | 18,866 |
| N90 | 6,226 | 7,033 |
| Mean Length | 11,924 | 13,380 |
| Max Length | 137,470 | 137,415 |

Table S3. The raw data information of RNA- sequencing.

| Sample | Raw reads | Total bases of raw data | Clean reads | Clean bases | GC content(%) | Total mapped | Multiple mapped | Uniquely mapped |
| --- | --- | --- | --- | --- | --- | --- | --- | --- |
| Mon | 21395915 | - | 20876612 | 6,208,784,986 | 52.02 | - | - | - |
| F1 | 55458558 | 8374242258 | 55103758 | 8,221,461,038 | 52.00 | 50978408(92.51%) | 2045796(3.71%) | 48932612(88.8%) |
| F2 | 49960440 | 7544026440 | 49560440 | 7,389,074,750 | 51.84 | 45612147(92.03%) | 1851576(3.74%) | 43760571(88.3%) |
| F3 | 56606312 | 8547553112 | 56203088 | 8,367222043 | 51.93 | 51468588(91.58%) | 2104841(3.75%) | 49363747(87.83%) |
| P1 | 53438220 | 8069171220 | 53001766 | 7,908384896 | 51.87 | 48764377(92.01%) | 2101141(3.96%) | 46663236(88.04%) |
| P2 | 53240076 | 8039251476 | 52815320 | 7,876629710 | 52.01 | 48761962(92.33%) | 2039948(3.86%) | 46722014(88.46%) |
| P3 | 49887448 | 7533004648 | 49531682 | 7,379135207 | 52.08 | 45393713(91.65%) | 2022285(4.08%) | 43371428(87.56%) |
| S1 | 48748818 | 7361071518 | 48253124 | 7,211219541 | 51.91 | 42277441(87.62%) | 2216037(4.59%) | 40061404(83.02%) |
| S2 | 49652108 | 7497468308 | 49106738 | 7,338489826 | 52.09 | 44237577(90.08%) | 2305124(4.69%) | 41932453(85.39%) |
| S3 | 47344648 | 7149041848 | 46876694 | 6,987544020 | 52.05 | 42289715(90.21%) | 2126167(4.54%) | 40163548(85.68%) |

**Table S4. The CAZymes genes expressions in 3 stages.**

|  |  | **FPKM** | | | **Differential expression** | | | | | | | |
| --- | --- | --- | --- | --- | --- | --- | --- | --- | --- | --- | --- | --- |
| **Gene id** | **CAZymes** | **S** | **P** | **F** |  | | **SP** | | **PF** | | **SF** | |
| EVM0004344 | AA2 | 293.84 | 1391.67 | 473.57 | | up | | down | |  | |  |
| EVM0006441 | GT4 | 137.15 | 992.50 | 969.11 | | up | |  | | up | |  |
| EVM0006334 | GH128 | 923.36 | 867.38 | 1003.62 | |  | |  | |  | |  |
| EVM0001690 | AA3_2 | 372.98 | 298.65 | 153.19 | |  | |  | | down | |  |
| EVM0000906 | CBM50 | 239.88 | 288.51 | 535.66 | |  | |  | | up | |  |
| EVM0001819 | AA3 | 215.58 | 284.08 | 219.54 | |  | |  | |  | |  |
| EVM0002915 | CE10 | 299.20 | 276.39 | 255.86 | |  | |  | |  | |  |
| EVM0009264 | GH71 | 50.50 | 274.21 | 411.74 | | up | |  | | up | |  |
| EVM0005132 | GH16 | 175.22 | 250.42 | 94.57 | |  | | down | |  | |  |
| EVM0004757 | CE10 | 242.75 | 243.04 | 22.06 | |  | | down | | down | |  |
| EVM0008892 | GT2_Glyco_trans_2_3 | 243.90 | 222.15 | 660.98 | |  | | up | |  | |  |
| EVM0001887 | GT22 | 90.20 | 221.14 | 149.37 | | up | |  | |  | |  |
| EVM0009019 | GH16 | 284.50 | 200.67 | 288.46 | |  | |  | |  | |  |
| EVM0002929 | AA4 | 71.64 | 195.02 | 64.96 | | up | | down | |  | |  |
| EVM0003802 | PL35 | 171.92 | 194.98 | 319.90 | |  | |  | |  | |  |
| EVM0001103 | GH72 | 54.08 | 191.35 | 152.52 | | up | |  | | up | |  |
| EVM0001557 | GH17 | 351.71 | 178.24 | 185.14 | | down | |  | | down | |  |
| EVM0008000 | CE10 | 230.31 | 177.32 | 183.31 | |  | |  | |  | |  |
| EVM0008985 | GH16 | 216.28 | 170.85 | 136.65 | |  | |  | |  | |  |
| EVM0004537 | GT3 | 94.29 | 157.63 | 91.02 | |  | |  | |  | |  |
| EVM0005860 | GH47 | 78.55 | 156.07 | 105.01 | |  | |  | |  | |  |
| EVM0007913 | CE4 | 157.45 | 153.65 | 65.43 | |  | | down | | down | |  |
| EVM0006103 | GT2_Chitin_synth_1 | 319.83 | 151.31 | 131.87 | | down | |  | | down | |  |
| EVM0006250 | GH5_9 | 119.35 | 144.66 | 137.78 | |  | |  | |  | |  |
| EVM0006667 | AA5_1 | 532.96 | 140.47 | 310.52 | | down | |  | |  | |  |
| EVM0002247 | GT66 | 33.16 | 138.14 | 57.89 | | up | | down | |  | |  |
| EVM0000973 | GT39 | 40.44 | 129.86 | 102.49 | | up | |  | | up | |  |
| EVM0003083 | AA9 | 204.79 | 129.63 | 92.29 | |  | |  | | down | |  |
| EVM0000221 | GH16 | 265.79 | 129.20 | 166.31 | | down | |  | |  | |  |
| EVM0001760 | GT2_Glycos_transf_2 | 32.45 | 127.73 | 43.76 | | up | | down | |  | |  |
| EVM0009178 | AA6 | 187.93 | 103.21 | 60.87 | | down | |  | | down | |  |
| EVM0008972 | GH37 | 141.78 | 96.39 | 90.60 | |  | |  | |  | |  |
| EVM0008428 | GH5_12 | 21.86 | 94.20 | 86.67 | | up | |  | | up | |  |
| EVM0006105 | GT20 | 85.04 | 88.66 | 77.81 | |  | |  | |  | |  |
| EVM0003664 | AA14.phmm | 36.13 | 88.57 | 50.64 | | up | |  | |  | |  |
| EVM0008301 | GH63 | 69.41 | 88.23 | 75.40 | |  | |  | |  | |  |
| EVM0007727 | AA3_2 | 55.33 | 88.20 | 57.83 | |  | |  | |  | |  |
| EVM0002528 | GH55 | 86.86 | 83.61 | 223.65 | |  | |  | | up | |  |
| EVM0007649 | GT8 | 98.49 | 82.68 | 468.40 | |  | | up | | up | |  |
| EVM0003390 | GT2_Chitin_synth_1 | 285.09 | 79.72 | 321.64 | | down | | up | |  | |  |
| EVM0005697 | GT15 | 69.65 | 78.88 | 141.31 | |  | |  | |  | |  |
| EVM0007809 | GT39 | 43.09 | 78.20 | 67.75 | |  | |  | |  | |  |
| EVM0000028 | GT28 | 207.47 | 75.90 | 156.94 | | down | |  | |  | |  |
| EVM0007691 | CE10 | 76.20 | 74.76 | 76.49 | |  | |  | |  | |  |
| EVM0008986 | PL8_4 | 24.40 | 71.97 | 44.31 | | up | |  | |  | |  |
| EVM0000838 | GH18 | 500.09 | 71.25 | 1097.48 | | down | | up | |  | |  |
| EVM0003868 | GH16 | 73.29 | 68.44 | 52.20 | |  | |  | |  | |  |
| EVM0006415 | GH31 | 31.00 | 67.98 | 51.06 | | up | |  | |  | |  |
| EVM0004845 | CE1 | 40.62 | 65.51 | 70.07 | |  | |  | |  | |  |
| EVM0003290 | GT2_Chitin_synth_2 | 75.04 | 65.17 | 80.90 | |  | |  | |  | |  |
| EVM0006491 | CE16 | 65.60 | 64.43 | 76.66 | |  | |  | |  | |  |
| EVM0001752 | GT69 | 33.30 | 63.79 | 46.66 | |  | |  | |  | |  |
| EVM0000256 | GH16 | 1230.55 | 63.20 | 6346.44 | | down | | up | | up | |  |
| EVM0008543 | AA7 | 67.14 | 63.18 | 70.14 | |  | |  | |  | |  |
| EVM0008900 | CE10 | 61.44 | 63.02 | 47.91 | |  | |  | |  | |  |
| EVM0007048 | CE10 | 61.44 | 63.02 | 47.90 | |  | |  | |  | |  |
| EVM0002848 | GH3 | 20.03 | 62.23 | 55.05 | | up | |  | |  | |  |
| EVM0007800 | GH29 | 42.56 | 62.09 | 133.40 | |  | | up | | up | |  |
| EVM0004692 | GH95 | 28.10 | 61.73 | 24.58 | |  | | down | |  | |  |
| EVM0004041 | CE10 | 63.26 | 59.99 | 99.45 | |  | |  | |  | |  |
| EVM0002873 | GH125 | 51.18 | 59.66 | 49.13 | |  | |  | |  | |  |
| EVM0006855 | GT2_Chitin_synth_2 | 63.65 | 59.55 | 88.91 | |  | |  | |  | |  |
| EVM0001383 | GT21 | 7.56 | 59.06 | 34.46 | | up | |  | | up | |  |
| EVM0007156 | GT2_Glycos_transf_2 | 73.16 | 56.64 | 81.05 | |  | |  | |  | |  |
| EVM0001873 | GH38 | 48.89 | 56.14 | 59.25 | |  | |  | |  | |  |
| EVM0003441 | CE10 | 44.17 | 55.71 | 60.26 | |  | |  | |  | |  |
| EVM0001716 | CE12 | 39.42 | 55.61 | 81.32 | |  | |  | |  | |  |
| EVM0005530 | GT39 | 58.38 | 55.54 | 50.95 | |  | |  | |  | |  |
| EVM0002857 | GH5_12 | 18.49 | 54.69 | 34.76 | | up | |  | |  | |  |
| EVM0007514 | CBM5 | 86.38 | 51.44 | 28.72 | |  | |  | | down | |  |
| EVM0003545 | CE10 | 33.69 | 50.35 | 24.46 | |  | |  | |  | |  |
| EVM0005978 | GT22 | 21.35 | 49.58 | 39.30 | | up | |  | |  | |  |
| EVM0002680 | GT48 | 93.24 | 49.56 | 70.36 | | down | |  | |  | |  |
| EVM0005514 | AA3 | 61.90 | 47.48 | 26.84 | |  | |  | | down | |  |
| EVM0005727 | GH27 | 9.57 | 47.42 | 152.70 | | up | | up | | up | |  |
| EVM0000957 | AA3_3 | 110.40 | 46.95 | 79.24 | | down | |  | |  | |  |
| EVM0003710 | GH9 | 11.68 | 46.76 | 23.71 | | up | |  | |  | |  |
| EVM0007195 | AA7 | 22.53 | 45.35 | 24.21 | |  | |  | |  | |  |
| EVM0002256 | GT1 | 35.95 | 44.61 | 37.14 | |  | |  | |  | |  |
| EVM0001116 | AA7 | 35.99 | 44.14 | 14.80 | |  | | down | | down | |  |
| EVM0003372 | GT2_Chitin_synth_2 | 23.37 | 44.07 | 34.52 | |  | |  | |  | |  |
| EVM0006980 | AA6 | 447.79 | 43.71 | 21.42 | | down | |  | | down | |  |
| EVM0004682 | CBM13 | 1872.75 | 43.55 | 294.16 | | down | | up | | down | |  |
| EVM0005809 | GT59 | 18.20 | 43.51 | 61.09 | |  | |  | | up | |  |
| EVM0006862 | GH3 | 27.80 | 43.43 | 28.19 | |  | |  | |  | |  |
| EVM0003870 | AA3_2 | 267.35 | 43.04 | 33.42 | | down | |  | | down | |  |
| EVM0007327 | AA7 | 55.39 | 42.22 | 140.61 | |  | | up | |  | |  |
| EVM0008474 | GT2_Chitin_synth_2 | 77.89 | 41.77 | 62.04 | | down | |  | |  | |  |
| EVM0001437 | GH20 | 9.82 | 41.45 | 23.52 | | up | |  | | up | |  |
| EVM0001143 | GH16 | 129.82 | 41.42 | 57.67 | | down | |  | | down | |  |
| EVM0002631 | GT90 | 22.50 | 40.60 | 26.25 | |  | |  | |  | |  |
| EVM0006223 | AA1_1 | 69.21 | 40.24 | 10.26 | |  | | down | | down | |  |
| EVM0009016 | CE10 | 21.79 | 39.94 | 13.48 | |  | |  | |  | |  |
| EVM0006791 | GT48 | 66.03 | 39.32 | 77.04 | |  | |  | |  | |  |
| EVM0000764 | GH13_8 | 38.00 | 38.84 | 55.96 | |  | |  | |  | |  |
| EVM0004345 | GT90 | 36.89 | 38.41 | 100.75 | |  | |  | |  | |  |
| EVM0003871 | GH79 | 23.15 | 38.21 | 10.12 | |  | | down | |  | |  |
| EVM0003945 | GH92 | 23.61 | 37.99 | 46.88 | |  | |  | |  | |  |
| EVM0007466 | GT2_Chitin_synth_2 | 173.73 | 37.63 | 92.81 | | down | | up | | down | |  |
| EVM0002037 | GH31 | 63.34 | 37.62 | 399.63 | |  | | up | | up | |  |
| EVM0001550 | CE10 | 18.83 | 36.86 | 53.16 | |  | |  | |  | |  |
| EVM0009276 | GT2_Glyco_tranf_2_3 | 47.84 | 36.66 | 73.96 | |  | |  | |  | |  |
| EVM0004207 | GT69 | 24.79 | 36.55 | 27.07 | |  | |  | |  | |  |
| EVM0008613 | GT4 | 16.86 | 36.27 | 18.47 | |  | | down | |  | |  |
| EVM0004390 | CE16 | 12.60 | 36.01 | 38.75 | | up | |  | |  | |  |
| EVM0005273 | GH76 | 3.19 | 35.93 | 4.27 | | up | |  | |  | |  |
| EVM0004855 | GT4 | 15.51 | 35.43 | 21.58 | |  | |  | |  | |  |
| EVM0001371 | GT8 | 7.43 | 34.58 | 18.57 | |  | |  | |  | |  |
| EVM0002291 | GT4 | 15.88 | 34.21 | 48.31 | | up | |  | | up | |  |
| EVM0003002 | GH13_22 | 99.57 | 33.82 | 48.08 | | down | |  | | down | |  |
| EVM0008750 | AA3_2 | 9.36 | 33.56 | 68.21 | | up | | up | | up | |  |
| EVM0007138 | GH35 | 42.21 | 33.25 | 31.55 | |  | |  | |  | |  |
| EVM0004067 | CBM13 | 20.98 | 33.17 | 15.52 | |  | |  | |  | |  |
| EVM0008826 | CBM13 | 1168.78 | 33.07 | 185.73 | | down | | up | | down | |  |
| EVM0005624 | AA7 | 256.58 | 32.80 | 185.18 | |  | |  | |  | |  |
| EVM0003896 | GH47 | 16.99 | 31.56 | 27.49 | |  | |  | |  | |  |
| EVM0004821 | CE10 | 21.38 | 31.35 | 29.90 | |  | |  | |  | |  |
| EVM0008916 | GH18 | 55.27 | 31.15 | 53.98 | | down | |  | |  | |  |
| EVM0000198 | GH13_5 | 15.06 | 30.86 | 35.40 | |  | |  | | up | |  |
| EVM0008118 | GT2_Chitin_synth_1 | 5.91 | 30.76 | 10.26 | | up | | down | |  | |  |
| EVM0007759 | GT57 | 19.52 | 30.58 | 25.44 | |  | |  | |  | |  |
| EVM0008998 | GH5_30 | 338.62 | 30.40 | 190.28 | | down | | up | |  | |  |
| EVM0008649 | GT24 | 14.09 | 30.33 | 19.65 | |  | |  | |  | |  |
| EVM0002244 | GH3 | 25.15 | 29.79 | 105.70 | |  | | up | | up | |  |
| EVM0005590 | GH37 | 122.15 | 29.71 | 7.03 | | down | | down | | down | |  |
| EVM0000380 | GH13_25 | 36.85 | 29.54 | 32.76 | |  | |  | |  | |  |
| EVM0009399 | GT2_Chitin_synth_1 | 65.63 | 29.30 | 30.05 | | down | |  | | down | |  |
| EVM0003613 | GH85 | 19.74 | 29.30 | 34.90 | |  | |  | |  | |  |
| EVM0001529 | GT20 | 47.39 | 28.72 | 34.20 | |  | |  | |  | |  |
| EVM0000044 | GT32 | 275.59 | 28.63 | 13.93 | | down | |  | | down | |  |
| EVM0005797 | GH47 | 39.34 | 27.04 | 86.43 | |  | | up | |  | |  |
| EVM0002695 | GT31 | 19.38 | 26.56 | 34.45 | |  | |  | |  | |  |
| EVM0008621 | GH15 | 36.21 | 25.74 | 20.79 | |  | |  | |  | |  |
| EVM0001434 | GT8 | 8.13 | 25.09 | 7.60 | | up | | down | |  | |  |
| EVM0009273 | CE4 | 2.52 | 24.93 | 14.65 | | up | |  | | up | |  |
| EVM0004473 | GT76 | 20.99 | 24.60 | 72.09 | |  | | up | | up | |  |
| EVM0004508 | GT22 | 16.53 | 23.25 | 32.31 | |  | |  | |  | |  |
| EVM0005859 | GH13_1 | 8.95 | 23.08 | 528.42 | | up | | up | | up | |  |
| EVM0007284 | AA1_1 | 31.14 | 22.99 | 29.29 | |  | |  | |  | |  |
| EVM0003583 | AA7 | 49.45 | 22.98 | 23.71 | | down | |  | | down | |  |
| EVM0008048 | CE10 | 45.19 | 22.48 | 67.18 | | down | | up | |  | |  |
| EVM0009419 | GH27 | 50.59 | 22.20 | 109.02 | |  | | up | | up | |  |
| EVM0005079 | GH16 | 6.08 | 21.82 | 24.33 | | up | |  | | up | |  |
| EVM0008600 | AA7 | 2.10 | 21.74 | 2.44 | | up | | down | |  | |  |
| EVM0006547 | AA3_3 | 31.20 | 21.46 | 32.71 | |  | |  | |  | |  |
| EVM0000917 | GT15 | 52.67 | 20.76 | 32.24 | |  | |  | |  | |  |
| EVM0008400 | GH79 | 20.95 | 20.71 | 9.98 | |  | | down | | down | |  |
| EVM0006707 | CE10 | 13.72 | 20.64 | 13.17 | |  | |  | |  | |  |
| EVM0006750 | PL14_4 | 22.58 | 20.64 | 8.13 | |  | | down | | down | |  |
| EVM0007723 | GH15 | 136.03 | 20.60 | 53.66 | | down | | up | | down | |  |
| EVM0006904 | GH92 | 17.23 | 20.40 | 32.91 | |  | |  | |  | |  |
| EVM0003019 | CE9 | 68.30 | 19.91 | 57.60 | | down | | up | |  | |  |
| EVM0007112 | GT20 | 147.03 | 19.83 | 14.73 | | down | |  | | down | |  |
| EVM0002585 | CE10 | 12.13 | 19.75 | 13.67 | |  | |  | |  | |  |
| EVM0003286 | GH5_9 | 27.09 | 19.39 | 46.74 | |  | | up | |  | |  |
| EVM0003387 | GH16 | 23.35 | 19.29 | 20.82 | |  | |  | |  | |  |
| EVM0004031 | GH31 | 9.15 | 19.25 | 15.01 | |  | |  | |  | |  |
| EVM0006147 | CE10 | 37.88 | 19.13 | 18.95 | | down | |  | | down | |  |
| EVM0002212 | CE10 | 22.81 | 18.70 | 24.31 | |  | |  | |  | |  |
| EVM0004670 | CE10 | 111.94 | 18.57 | 15.53 | | down | |  | | down | |  |
| EVM0000867 | GT33 | 17.59 | 18.48 | 20.15 | |  | |  | |  | |  |
| EVM0002724 | GT22 | 8.68 | 18.41 | 14.61 | |  | |  | |  | |  |
| EVM0003016 | GH20 | 20.79 | 18.19 | 26.49 | |  | |  | |  | |  |
| EVM0004237 | GT57 | 11.80 | 17.79 | 22.50 | |  | |  | |  | |  |
| EVM0004525 | GH15 | 9.31 | 17.37 | 5.17 | |  | |  | |  | |  |
| EVM0003971 | GH5_15 | 102.04 | 17.20 | 381.94 | | down | | up | |  | |  |
| EVM0004359 | GH88 | 13.43 | 16.95 | 21.46 | |  | |  | |  | |  |
| EVM0009029 | GH43_30 | 51.62 | 16.89 | 1.67 | |  | | down | | down | |  |
| EVM0001035 | AA9 | 38.35 | 16.73 | 10.97 | | down | |  | | down | |  |
| EVM0007558 | AA7 | 40.28 | 16.46 | 35.22 | | down | |  | |  | |  |
| EVM0006282 | AA14.phmm | 2.90 | 16.26 | 9.17 | | up | |  | |  | |  |
| EVM0002211 | GH47 | 20.60 | 16.09 | 19.58 | |  | |  | |  | |  |
| EVM0003757 | CE10 | 2.74 | 15.58 | 83.75 | | up | | up | | up | |  |
| EVM0004552 | AA7 | 13.72 | 15.47 | 16.06 | |  | |  | |  | |  |
| EVM0002384 | GH18 | 28.44 | 15.45 | 18.01 | | down | |  | |  | |  |
| EVM0008329 | AA3_2 | 28.92 | 15.34 | 16.36 | |  | |  | |  | |  |
| EVM0005602 | CE10 | 8.65 | 15.29 | 8.84 | |  | |  | |  | |  |
| EVM0007229 | CE10 | 8.65 | 15.29 | 8.84 | |  | |  | |  | |  |
| EVM0005732 | AA1_1 | 49.19 | 14.93 | 3.41 | | down | | down | | down | |  |
| EVM0008143 | GH16 | 230.05 | 14.91 | 23.20 | | down | |  | | down | |  |
| EVM0003715 | CE10 | 22.25 | 14.90 | 34.75 | |  | |  | |  | |  |
| EVM0000841 | AA3_3 | 14.55 | 14.60 | 9.90 | |  | |  | |  | |  |
| EVM0001345 | GH105 | 21.80 | 14.45 | 19.42 | |  | |  | |  | |  |
| EVM0005974 | GH79 | 23.63 | 14.44 | 16.99 | |  | |  | |  | |  |
| EVM0006201 | CE10 | 3.98 | 14.24 | 4.61 | | up | | down | |  | |  |
| EVM0000275 | GH25 | 23.14 | 13.86 | 19.93 | |  | |  | |  | |  |
| EVM0005384 | AA9 | 19.51 | 13.66 | 17.97 | |  | |  | |  | |  |
| EVM0009444 | GT49 | 2.57 | 13.62 | 12.13 | | up | |  | | up | |  |
| EVM0005387 | GH5_50 | 9.36 | 13.39 | 12.84 | |  | |  | |  | |  |
| EVM0009271 | GH31 | 12.30 | 13.24 | 13.38 | |  | |  | |  | |  |
| EVM0004989 | GH31 | 31.46 | 13.17 | 10.96 | | down | |  | | down | |  |
| EVM0007036 | GT35 | 46.41 | 12.95 | 88.77 | | down | | up | |  | |  |
| EVM0007406 | CE10 | 93.41 | 12.68 | 9.26 | | down | |  | | down | |  |
| EVM0005907 | GT15 | 17.59 | 12.63 | 17.19 | |  | |  | |  | |  |
| EVM0006774 | GH2 | 16.46 | 12.59 | 43.51 | |  | | up | | up | |  |
| EVM0006729 | GT4 | 5.44 | 11.60 | 6.48 | |  | |  | |  | |  |
| EVM0002460 | CE10 | 10.97 | 11.50 | 16.34 | |  | |  | |  | |  |
| EVM0004667 | CE10 | 22.10 | 11.47 | 30.18 | | down | | up | |  | |  |
| EVM0001516 | GT50 | 10.62 | 11.29 | 17.59 | |  | |  | |  | |  |
| EVM0008006 | GH71 | 12.56 | 11.22 | 14.49 | |  | |  | |  | |  |
| EVM0002731 | GT2_Glycos_transf_2 | 12.94 | 11.19 | 19.76 | |  | |  | |  | |  |
| EVM0009001 | GH31 | 8.97 | 11.05 | 21.33 | |  | |  | | up | |  |
| EVM0004616 | CE10 | 2.89 | 11.00 | 4.84 | | up | | down | |  | |  |
| EVM0009036 | GT69 | 24.97 | 10.98 | 13.46 | | down | |  | | down | |  |
| EVM0006975 | GH89 | 54.59 | 10.95 | 55.25 | | down | | up | |  | |  |
| EVM0005610 | PL14_5 | 18.28 | 10.88 | 45.16 | |  | | up | | up | |  |
| EVM0000539 | GH16 | 142.40 | 10.63 | 9.44 | | down | |  | | down | |  |
| EVM0009188 | AA7 | 7.90 | 10.57 | 10.42 | |  | |  | |  | |  |
| EVM0002119 | AA3_2 | 4.18 | 10.33 | 13.80 | |  | |  | | up | |  |
| EVM0001572 | GH47 | 10.90 | 10.15 | 19.58 | |  | |  | |  | |  |
| EVM0003282 | AA1_1 | 4.07 | 10.13 | 10.80 | |  | |  | |  | |  |
| EVM0009027 | AA1_2 | 8.73 | 10.02 | 15.04 | |  | |  | |  | |  |
| EVM0000834 | GH5_12 | 0.00 | 9.73 | 6.88 | | up | |  | | up | |  |
| EVM0007681 | CE10 | 4.29 | 9.63 | 6.86 | | up | |  | |  | |  |
| EVM0001870 | GH18 | 7.68 | 9.62 | 71.25 | |  | | up | | up | |  |
| EVM0002464 | GH27 | 10.98 | 9.61 | 10.80 | |  | |  | |  | |  |
| EVM0007873 | AA1_1 | 10.30 | 9.53 | 20.52 | |  | | up | |  | |  |
| EVM0007838 | GH105 | 8.84 | 9.46 | 15.83 | |  | |  | |  | |  |
| EVM0001718 | GH16 | 10.04 | 8.99 | 17.60 | |  | |  | |  | |  |
| EVM0006974 | GH18 | 6.31 | 8.74 | 11.88 | |  | |  | |  | |  |
| EVM0002623 | CBM13 | 91.96 | 8.56 | 19.90 | | down | | up | | down | |  |
| EVM0008500 | AA7 | 7.94 | 8.46 | 9.80 | |  | |  | |  | |  |
| EVM0003349 | AA5_1 | 7.81 | 8.22 | 7.14 | |  | |  | |  | |  |
| EVM0000783 | GH16 | 26.02 | 7.97 | 8.44 | | down | |  | | down | |  |
| EVM0001708 | GH30_3 | 12.82 | 7.66 | 12.10 | |  | |  | |  | |  |
| EVM0008317 | CE14 | 5.45 | 7.53 | 11.09 | |  | |  | |  | |  |
| EVM0006537 | GH128 | 0.30 | 7.49 | 18.47 | | up | |  | | up | |  |
| EVM0006681 | AA1_1 | 6.44 | 7.45 | 16.01 | |  | | up | | up | |  |
| EVM0004275 | GH18 | 9.19 | 7.42 | 17.80 | |  | | up | |  | |  |
| EVM0000033 | GH128 | 5.75 | 6.94 | 13.03 | |  | |  | |  | |  |
| EVM0004811 | CE10 | 5.74 | 6.91 | 15.48 | |  | |  | | up | |  |
| EVM0004285 | AA1_1 | 94.58 | 6.90 | 7.01 | | down | |  | | down | |  |
| EVM0002554 | CE10 | 2.54 | 6.85 | 2.89 | | up | | down | |  | |  |
| EVM0004943 | CE16 | 0.45 | 6.76 | 1.16 | | up | |  | |  | |  |
| EVM0000956 | GH29 | 7.63 | 6.52 | 7.11 | |  | |  | |  | |  |
| EVM0009347 | GH16 | 5.03 | 6.45 | 9.63 | |  | |  | |  | |  |
| EVM0003023 | GH31 | 0.75 | 6.34 | 2.50 | | up | | down | | up | |  |
| EVM0003685 | AA1_2 | 3.68 | 6.21 | 21.29 | |  | | up | | up | |  |
| EVM0009268 | AA7 | 12.19 | 6.08 | 8.15 | |  | |  | |  | |  |
| EVM0007703 | GH31 | 2.66 | 5.79 | 6.03 | | up | |  | |  | |  |
| EVM0009414 | AA1_1 | 5.50 | 5.56 | 10.14 | |  | |  | |  | |  |
| EVM0002762 | GH16 | 107.59 | 5.23 | 0.40 | | down | | down | | down | |  |
| EVM0006348 | GH13_40 | 25.32 | 5.20 | 11.27 | | down | |  | | down | |  |
| EVM0002902 | GH89 | 2.38 | 5.04 | 7.28 | | up | |  | | up | |  |
| EVM0008629 | AA3 | 9.06 | 4.86 | 7.97 | |  | |  | |  | |  |
| EVM0004435 | CE10 | 8.54 | 4.82 | 6.60 | |  | |  | |  | |  |
| EVM0005902 | AA9 | 74.04 | 4.81 | 16.19 | | down | |  | | down | |  |
| EVM0004854 | CE10 | 5.53 | 4.79 | 6.25 | |  | |  | |  | |  |
| EVM0008931 | AA1_1 | 1.62 | 4.73 | 1.45 | |  | |  | |  | |  |
| EVM0001863 | CE10 | 2.62 | 4.63 | 7.36 | |  | |  | |  | |  |
| EVM0003243 | AA7 | 5.27 | 4.42 | 3.54 | |  | |  | |  | |  |
| EVM0000895 | GH27 | 5.63 | 4.35 | 6.59 | |  | |  | |  | |  |
| EVM0001714 | GH13_32 | 10.75 | 4.22 | 19.87 | | down | | up | |  | |  |
| EVM0002770 | AA1_1 | 118.32 | 4.22 | 6.99 | | down | |  | | down | |  |
| EVM0004872 | GH55 | 8.15 | 4.07 | 23.24 | |  | | up | |  | |  |
| EVM0001851 | AA3_3 | 7.44 | 3.91 | 7.20 | |  | |  | |  | |  |
| EVM0000955 | GH13_1 | 3.06 | 3.79 | 8.23 | |  | |  | |  | |  |
| EVM0000260 | AA7 | 3.38 | 3.66 | 5.38 | |  | |  | |  | |  |
| EVM0005879 | CBM5 | 5.10 | 3.63 | 9.77 | |  | | up | |  | |  |
| EVM0006488 | CBM5 | 2.19 | 3.48 | 4.82 | |  | |  | |  | |  |
| EVM0006812 | AA5_1 | 1.72 | 3.10 | 4.06 | |  | |  | | up | |  |
| EVM0001373 | CE2 | 5.09 | 3.01 | 7.09 | |  | |  | |  | |  |
| EVM0005867 | GH18 | 2.13 | 2.86 | 10.79 | |  | | up | | up | |  |
| EVM0001357 | PL14 | 0.76 | 2.82 | 6.51 | |  | |  | | up | |  |
| EVM0007604 | GT58 | 0.00 | 2.73 | 4.93 | | up | |  | |  | |  |
| EVM0002128 | CE16 | 58.45 | 2.73 | 109.30 | | down | | up | |  | |  |
| EVM0008913 | CBM13 | 6.31 | 2.72 | 12.50 | | down | | up | |  | |  |
| EVM0007507 | AA9 | 1.30 | 2.55 | 6.89 | |  | |  | | up | |  |
| EVM0001296 | GH23 | 0.00 | 2.40 | 8.13 | | up | |  | | up | |  |
| EVM0008527 | CE10 | 4.20 | 2.40 | 6.19 | |  | | up | |  | |  |
| EVM0000331 | GH5_9 | 10.55 | 2.38 | 4.19 | | down | |  | | down | |  |
| EVM0002672 | GH18 | 0.74 | 2.25 | 0.74 | | up | | down | |  | |  |
| EVM0003035 | CBM5 | 1.24 | 2.22 | 4.06 | |  | |  | | up | |  |
| EVM0006572 | GH16 | 1.85 | 1.95 | 2.17 | |  | |  | |  | |  |
| EVM0004198 | AA1_1 | 2.18 | 1.94 | 4.95 | |  | | up | |  | |  |
| EVM0003071 | CBM5 | 24.88 | 1.93 | 27.68 | | down | | up | |  | |  |
| EVM0004965 | GT8 | 23.53 | 1.91 | 6.17 | | down | | up | | down | |  |
| EVM0001209 | AA7 | 2.69 | 1.85 | 6.93 | |  | | up | |  | |  |
| EVM0007513 | GH152 | 4.47 | 1.73 | 2.79 | |  | |  | |  | |  |
| EVM0007995 | CBM5 | 1.14 | 1.61 | 2.54 | |  | |  | |  | |  |
| EVM0000519 | PL14 | 0.93 | 1.53 | 6.19 | |  | | up | | up | |  |
| EVM0008799 | AA1_1 | 15.60 | 1.51 | 5.44 | | down | | up | | down | |  |
| EVM0004744 | CBM21 | 115.03 | 1.47 | 2.31 | | down | |  | | down | |  |
| EVM0008732 | GH71 | 3.04 | 1.40 | 4.55 | |  | | up | |  | |  |
| EVM0008548 | AA7 | 1.96 | 1.37 | 3.64 | |  | |  | |  | |  |
| EVM0000046 | GH3 | 0.48 | 1.31 | 0.00 | |  | |  | |  | |  |
| EVM0009138 | CBM13 | 2.66 | 1.02 | 1.99 | | down | |  | |  | |  |
| EVM0009064 | GH152 | 0.22 | 0.82 | 0.45 | |  | |  | |  | |  |
| EVM0000614 | GT22 | 0.44 | 0.45 | 2.10 | |  | |  | |  | |  |
| EVM0007932 | CE10 | 0.10 | 0.04 | 0.04 | |  | |  | |  | |  |
| EVM0005069 | CE10 | 0.02 | 0.03 | 0.03 | |  | |  | |  | |  |
| EVM0008284 | GH20 | 0.01 | 0.00 | 1.25 | |  | | up | | up | |  |
| EVM0009439 | GH20 | 0.01 | 0.00 | 1.25 | |  | | up | | up | |  |
| EVM0008150 | GH152 | 0.00 | 0.00 | 0.66 | |  | |  | |  | |  |
| EVM0005258 | GT25 | 0.00 | 0.00 | 0.00 | |  | |  | |  | |  |
| EVM0009202 | GH16 | 0.00 | 0.00 | 0.00 | |  | |  | |  | |  |


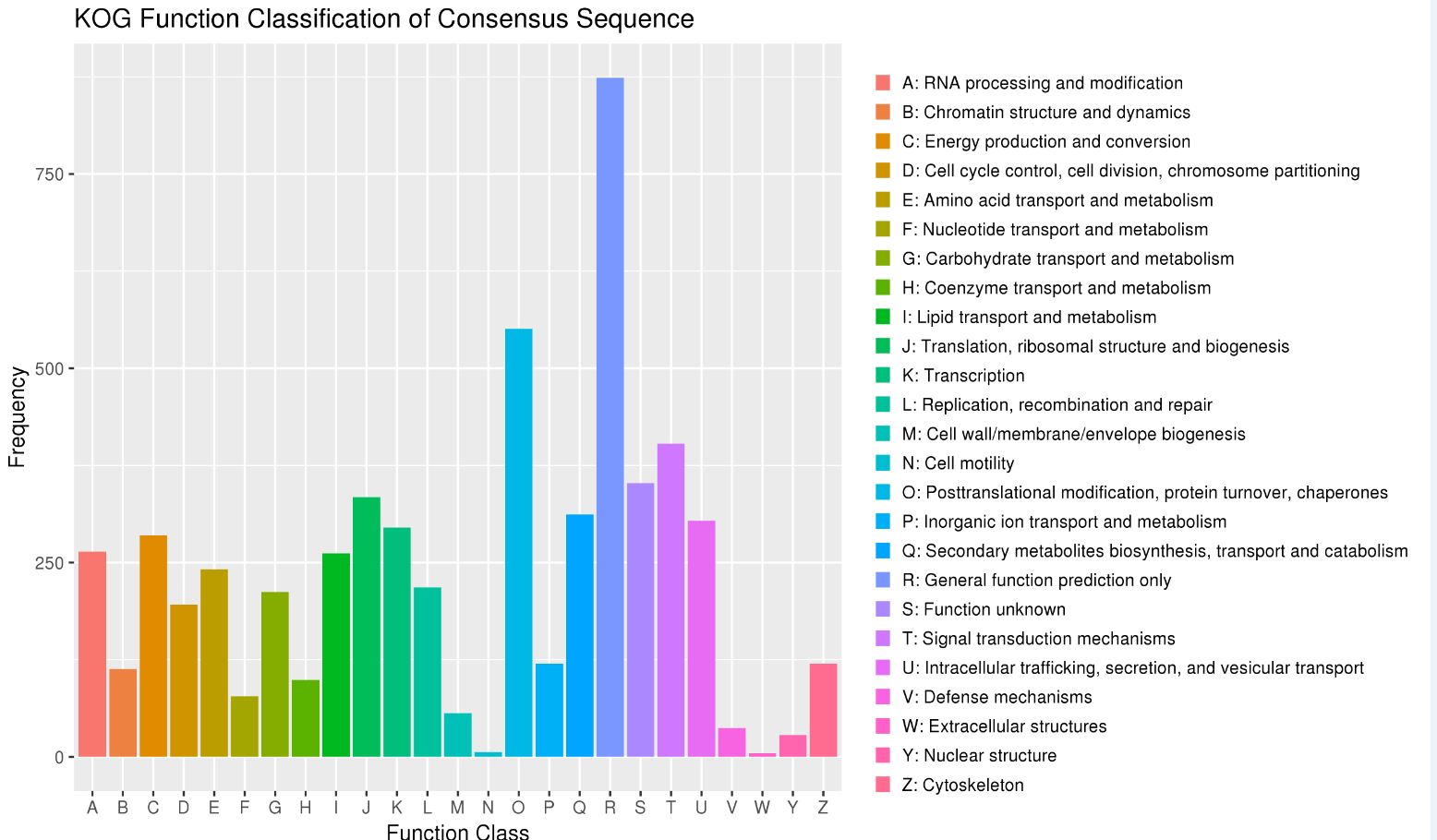


Figure S1. Gene annotation in the functional categorization using EuKaryotic Ortho-logous Group (KOG).


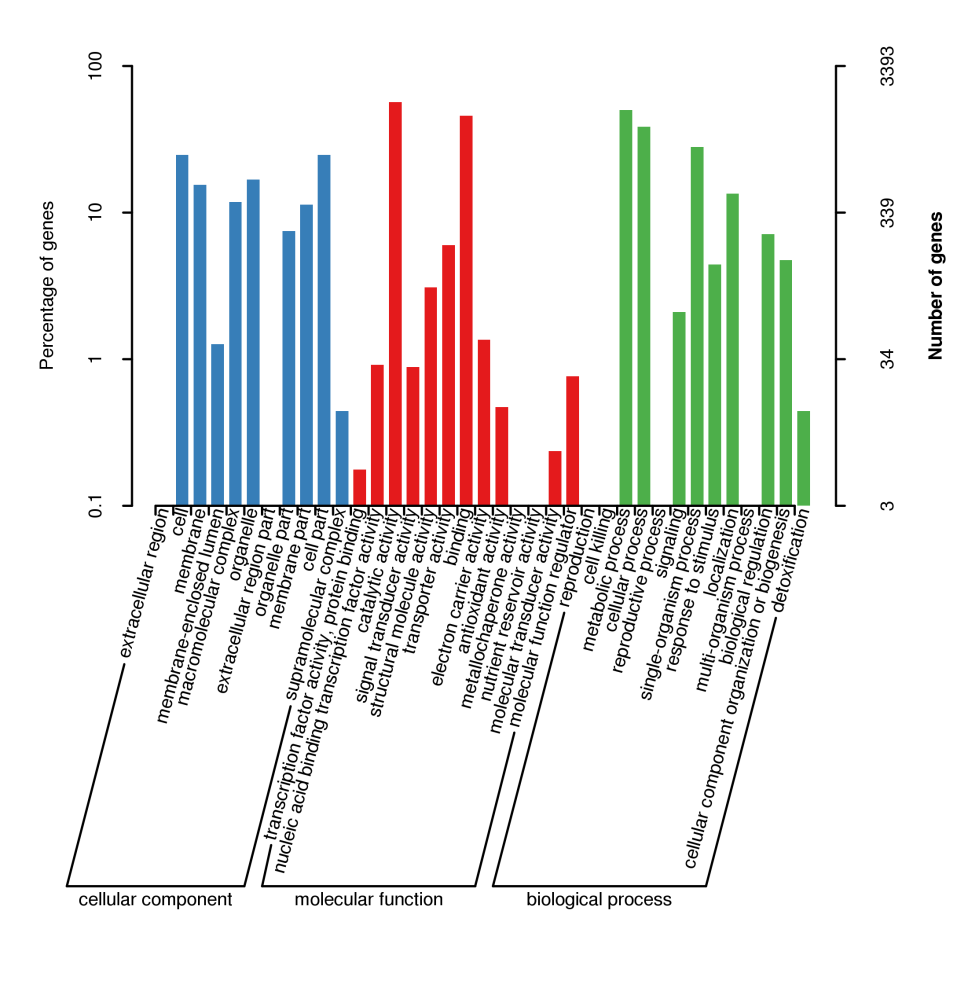


Figure S2. Gene ontology classification of the gene annotation the genome of *P. portentosus*


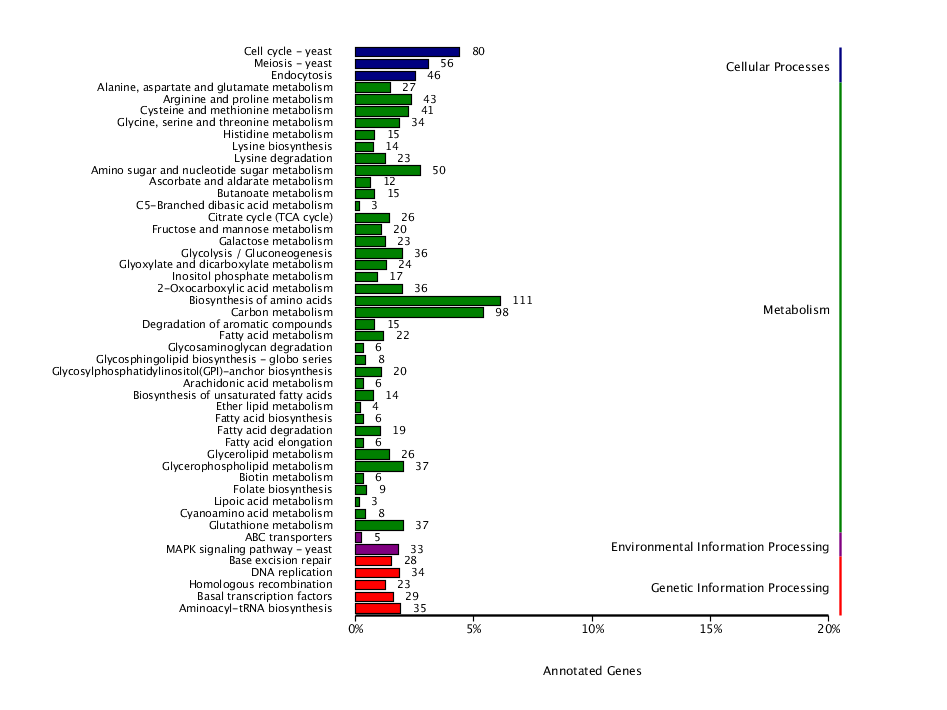


Figure S3. KEGG pathways annotated based on the KEGG database


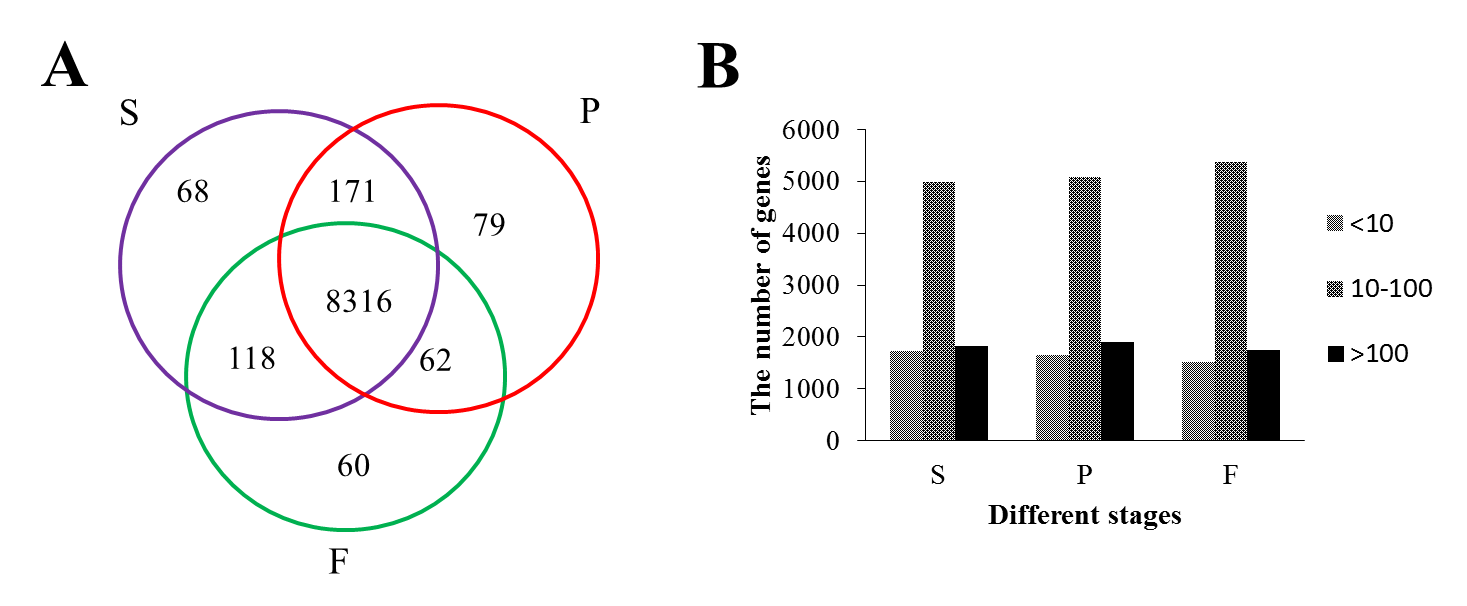


Figure S4. The gene expressions in different stages. A: Venn diagrams of shared gene expressions between different stages; B: three categories of the genes expressions in different stages based on FPKM values (FPKM <10, 10-100 and >100).


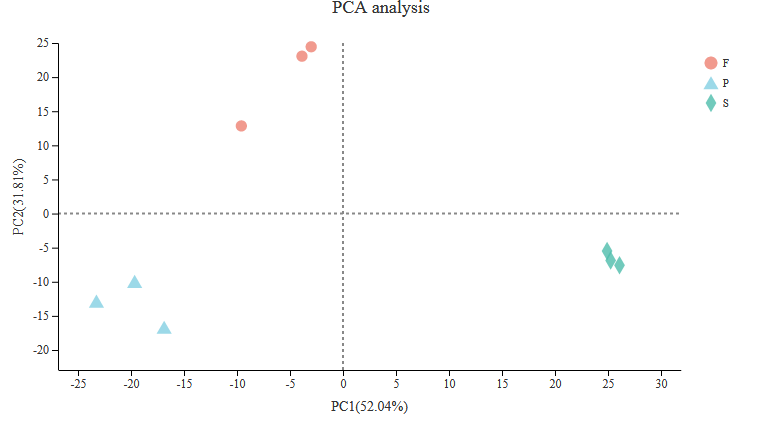


Figure S5. Principal component analysis based on FPKM values in different samples from three stages.
